# Comparative RNA-Seq Analyses of *Solenopsis japonica* (Hymenoptera: Formicidae) Reveal Gene in Response to Cold Stress

**DOI:** 10.3390/genes12101610

**Published:** 2021-10-13

**Authors:** Mohammad Vatanparast, Youngjin Park

**Affiliations:** Plant Quarantine Technology Center, Animal and Plant Quarantine Agency, Gimcheon 39660, Korea; mvatanparast@korea.kr

**Keywords:** *Solenopsis japonica*, transcriptome, KEGG pathway, differentially expressed gene, GO analysis, temperature, stress

## Abstract

*Solenopsis japonica*, as a fire ant species, shows some predatory behavior towards earthworms and woodlice, and preys on the larvae of other ant species by tunneling into a neighboring colony’s brood chamber. This study focused on the molecular response process and gene expression profiles of *S. japonica* to low (9 °C)-temperature stress in comparison with normal temperature (25 °C) conditions. A total of 89,657 unigenes (the clustered non-redundant transcripts that are filtered from the longest assembled contigs) were obtained, of which 32,782 were annotated in the NR (nonredundant protein) database with gene ontology (GO) terms, gene descriptions, and metabolic pathways. The results were 81 GO subgroups and 18 EggNOG (evolutionary genealogy of genes: Non-supervised Orthologous Groups) keywords. Differentially expressed genes (DEGs) with log_2_fold change (FC) > 1 and log_2_FC < −1 with *p*-value ≤ 0.05 were screened for cold stress temperature. We found 215 unigenes up-regulated and 115 unigenes down-regulated. Comparing transcriptome profiles for differential gene expression resulted in various DE proteins and genes, including fatty acid synthases and lipid metabolism, which have previously been reported to be involved in cold resistance. We verified the RNA-seq data by qPCR on 20 up- and down-regulated DEGs. These findings facilitate the basis for the future understanding of the adaptation mechanisms of *S. japonica* and the molecular mechanisms underlying the response to low temperatures.

## 1. Introduction

Fire ants is the common name for the ant genus *Solenopsis* Westwood (Hymenoptera: Formicidae: Myrmicinae) that is represented in South America by 16 native species [1]. While, in general, these ants cause occasional local problems in their homeland, some species, such as *Solenopsis japonica,* are not reported pests. With designing a new bait trap, it was clarified that *S. japonica* showed some predatory behavior against earthworms and woodlice [2] that have not previously been considered as predators of soil invertebrates. It is supposed that it preys on the larvae of other ant species by tunneling into a neighboring colony’s brood chamber [2]. Another article mentions that *S. japonica* lives near to the colonies of *Pheidole fervida*, *Rhizomyrma sauteri*, *Lasius flavus* and *Lasius niger* and builds subterranean tunnels leading into the nests of its neighbors [3]. *S. japonica* can move through gaps in the soil or make tunnel systems to detect and attack prey [2]. Unfortunately, there is not a massive amount of literature available on the biology of this ant species. Actually, species interactions underground cannot be observed directly, although they are suspected to occur as frequently as those above ground.

*S. japonica* is probably one of the influential underground predators that we have little evidence of their roles and effects on underground invertebrate communities [2,4].

The life history of insects, population dynamics, geographical distribution, species interactions, and community structure and function are affected by different temperatures, one of the vital abiotic factors, by interfering with their metabolic processes such as alimentation, digestion, detoxification, mating, growth, and life cycle, as well as the distribution of host plants [5,6,7,8,9,10,11,12,13]. Metabolic rates are directly tied to ambient temperatures in insects as ectotherms [14]. In the prey–predator relationship, attack rates by predators can be increased by increasing metabolic rates [15] which can increase predator interactions with prey, leading eventually to changes in the density of prey and a series of effects on wide community structure [16,17]. Many studies have repeatedly revealed that increased predation rates by invertebrate predators resulted from increased temperatures [15,18,19,20]. Ant *Formica lasioides* predation has been observed in laboratory conditions to be affected by temperature and that its prey, caterpillars of the tiger moth, *Arctia virginalis,* maintain activity and escape from ant predation at lower temperatures (8–14 °C) [19].

*S. japonica* as a soil dwelling invertebrate predator with a small body size (less than 2 mm) that would aid movement through the soil, can be considered as a beneficial predator. Previous studies have not indicated their capability, probably because their capabilities to move and attack prey in the soil have been overlooked.

In this study, we are looking for any changes under low and high temperature stresses using RNA-Seq and de novo transcriptome as an ‘omic’ technology to figure out which pathways are responsible for stress conditions and whether our model insect, *S. japonica,* can cope with that. There are some similar studies [21,22,23] to predict the regulation of transcription associated with different treatment temperatures.

Upregulation of cuticle genes [24] changes the expression levels of more than hundreds of genes associated with transcription, metabolism, and cuticular organization, especially enzyme-related genes as well as high-temperature stress, which upregulates encoding of cytochrome P450s (CYPs), antioxidative enzymes, and aldehyde dehydrogenase [21,25,26], and the expression of heat shock proteins (HSPs), reported to be responsible for heat and cold tolerance and other stressor as well (e.g., heat, low oxygen levels, UV radiation, bacterial and viral infection, heavy metals) that can affect the folding and functional conformation of proteins [27,28], is related to different ambient environmental temperature. In the current study, worker ants of *S. japonica* were incubated at a low temperature (9 °C). Detailed differential expression analysis revealed a number of candidate genes are potentially related to cold tolerance compared with 25 °C incubated ants of *S. japonica*. We confirmed our transcriptome data by performing qRT-PCR. We aimed to provide a basis for the adaptive mechanism and a rich resource for the discovery and identification of novel genes involved in the cold stress response in *S. japonica,* a rare underground-dwelling invertebrate predator.

## 2. Materials and Methods

### 2.1. Insect Rearing, Exposure Temperatures and Sample Preparation

An *S. japonica* colony was collected on Mt. Manin, Deajeon, Korea. The ant colony consists of dealate mated queens, alate queens, males, brood (eggs, larvae, and pupae), and workers. We used the field colony (colony of origin) to create standardized experimental colonies. Only the field colonies found to contain multiple queens were included in this experiment. All ants were placed in a plastic tray (25 (H) × 30 × 35 cm^3^) and maintained at 25 ± 1 °C in the Quarantine Pest Research Facility (Animal and Plant Quarantine Agency, Gimcheon, South Korea) with 65 ± 2% relative humidity. Ants were fed a 20% sucrose solution and mealworms (*Tenebrio molitor* larvae) as a protein source. Water was provided ad libitum. We generated the lab colony with single reproductive queens isolated from field-collected nests to obtain known offspring of individual queens from polygyne nests; the lab colony, comprising of a few thousand workers and brood, were maintained individually in plastic trays. We waited for six weeks before sampling pupae because some brood were the offspring of queens from the polygyne source nest other than the isolated queen, so that all such brood had eclosed as adults. In order to acquire known progeny, we also observed foraging behavior in standardized colonies in the lab, controlling for variation due to colony size and brood-to-worker ratio. To perform transcriptomic analysis, 15 *S. japonica* workers (body weight; 0.64 ± 0.22 mg) were incubated at 9 and 25 °C for 24 h as the temperature treatment groups. Incubated ants at 25 °C were considered as the control group. Each treatment was replicated with three independent biological sample preparations. After the temperature treatment, worker ants from each group were immediately frozen in liquid nitrogen and stored at −80 °C for subsequent experiments.

### 2.2. RNA Extraction and RT-qPCR

RNA samples were extracted from the whole bodies of *S. japonica* workers cast using Trizol reagent (Invitrogen, Carlsbad, CA, USA) according to the manufacturer’s instructions. After RNA extraction, it was resuspended in nuclease-free water and quantified using a spectrophotometer (NanoDrop, Thermo Scientific, Wilmington, DE, USA). cDNA was then synthesized from RNA (1 μg) using RT PreMix (Intron Biotechnology, Seoul, Korea) containing an oligo dT primer according to the manufacturer’s instructions. All quantitative PCRs (qPCRs) in this study were determined using a real-time PCR machine (CFX Connect real-time PCR Detection System, Bio-Rad, Hercules, CA, USA) and iQ SYBR Green Supermix (Bio-Rad, Hercules, CA, USA) according to the guidelines of the manufacturer. The reaction mixture (20 μL) contains 10 μL of iQ SYBR Green Supermix, 1 μL of cDNA template (100 ng), and 1 μL each of forward and reverse primers (Table S1) and 7 μL nuclease-free water. RT-qPCR cycling began with a 95 °C heat treatment for 10 min, followed by 40 cycles of denaturation at 94 °C for 30 s, annealing at 52 °C for 30 s, and extension at 72 °C for 20 s. The expression level of *rpl32* as a reference gene was used to normalize target gene expression levels [29] under different treatments. PCR products were assessed by melting curve analysis. Quantitative analysis was performed using the comparative CT (2^−ΔΔCT^) method [30].

### 2.3. Illumina Sequencing

To obtain short-read RNA sequences, Illumina sequencing was performed at Macrogen (Seoul, South Korea). Each library was constructed from 1 μg total RNA from the whole body of 5 individuals of *S. japonica* adults per treatment using the TruSeq Stranded mRNA LT Sample Prep Kit (Illumina, San Diego, CA, USA) and sequenced using the HiSeq 4000 System (Illumina, San Diego, CA, USA) with 101 bp pair end read (Table S2).

### 2.4. De Novo Assembly

Illumina short reads were quality-filtered and adapter-trimmed using Trimmomatic v0.38 (http://www.usadellab.org/cms/?page=trimmomatic, accessed on 2 July 2020). FastQC v0.11.7 (http://www.bioinformatics.babraham.ac.uk/projects/fastqc/, accessed on 2 July 2020) was used to check data quality before and after trimming. After the removal of low-quality reads, an Illumina-based de novo transcriptome assembly was performed using Trinity version trinity rnaseq r20140717, bowtie 1.1.2 [31]. Trimmed reads for every sample were merged into one file to construct a combined reference. The de novo assembly of merged data was carried out using Trinity with default parameters, and it was assembled into transcript contigs [32]. The total number of genes, transcripts, GC content, max/min/median/average contig length, and total assembled bases were summarized. Trinity groups transcripts into clusters based on shared sequence content. For assembled genes, the longest contigs of the assembled contigs are filtered and clustered into non-redundant transcripts using CD-HIT version 4.6 (http://weizhongli-lab.org/cd-hit, accessed on 2 July 2020) [33]. These transcripts were defined as ‘unigenes’, which are used for predicting Open Reading Frames (ORFs), annotating against several known sequence databases, and analyzing differentially expressed genes (DEGs). The ORF prediction for unigenes was performed using TransDecoder version 3.0.1 (https://github.com/TransDecoder/TransDecoder/wiki, accessed on 2 July 2020) [34] to identify candidate coding regions within the transcript sequence. After extracting ORFs that were at least 100 amino acids long, the TransDecoder predicted the likely coding regions. Trimmed reads for each sample were aligned to the assembled reference using the Bowtie program. For the differentially expressed gene analysis, the abundances of unigenes across samples were estimated into read count as an expression measure by the RSEM algorithm (RSEM version v1.2.29, bowtie 1.1.2, http://deweylab.github.io/RSEM/ (accessed on 2 July 2020) [35]).

### 2.5. Gene Functional Annotation

For functional annotation, unigenes were searched against the Kyoto Encyclopedia of Genes and Genomes (KEGG) v20190104 (http://www.genome.jp/kegg/ko.html, accessed on 2 July 2020), [36], NCBI Nucleotide (NT) v20180116 (https://www.ncbi.nlm.nih.gov/nucleotide/, accessed on 2 July 2020) [22], Pfam v20160316 (https://pfam.xfam.org/, accessed on 2 July 2020), [37], GO v20180319 (http://www.geneontology.org/, accessed on 2 July 2020), [38], NCBI NR v20180503 (https://www.ncbi.nlm.nih.gov/protein/, accessed on 2 July 2020), [39], UniProt v20180116 (http://www.uniprot.org/, accessed on 2 July 2020), [40] and EggNOG (http://eggnogdb.embl.de/, accessed on 2 July 2020) [41] using BLASTN of NCBI BLAST and BLASTX of DIAMOND version 0.9.21 (https://github.com/bbuchfink/diamond, accessed on 2 July 2020) with an *E*-value default cutoff of *E*-value 10^−5^.

### 2.6. Differential Gene Expression Analysis

A quality check was performed for all samples, so that if more than one read count value was 0, it was not included in the analysis. Gene expression levels were measured in by RNA-Seq analysis as the fragments per kilo base of transcript per million mapped reads (FPKM) [42]. Multiple testing was corrected for in all statistical tests using the Benjamini–Hochberg false discovery rate with the following parameter values: false discovery rate < 0.01 [43]. In order to reduce systematic bias, we estimate the size factors from the count data and apply Relative Log Expression normalization with the DESeq2 R library. Using each sample’s normalized value, the high expression similarities were grouped together by Hierarchical Clustering Analysis and graphically shown in a 2D plot to show the variability of the total data using Multidimensional Scaling Analysis. Significant unigene results were analyzed as up- and down-regulated count by log2FC ≥ 1 and ≤−1. The distribution of expression levels between the two groups was plotted as a Volcano plot and simple bar plots [44].

### 2.7. Quantitative RT-PCR Validation

A further twenty genes in response to cold treatment (T9) were chosen for validation using RT-qPCR. To choose the genes, the DEGs were sorted by fold change, and 10 DEGs were picked at random from the entire library for up-regulated DEGs and 10 DEGs for down-regulated DEGs. The DEGs that are annotated in all databases were chosen. *S. japonica* adults were incubated at 9 and 25 °C in two separated groups, including 10 ants, for 24 h. RNA was extracted from all ants together (a pool of 10 ants) and cDNA was synthesized according to the ‘RNA extraction and RT-qPCR’ section. Specific primers were designed using the Primer Quest tool (www.idtdna.com, accessed on 30 July 2020) (Table S1). To normalize target gene expression levels under various treatments, the expression level of *rpl32* was used as a reference gene [29]. Single PCR products were confirmed by 1% agarose gel and were assessed by melting curve analysis. Each treatment was replicated with three independent biological sample preparations. The comparative CT (2^−ΔΔCT^) method was used to perform quantitative analysis in three technical replicates [30]. Finally, the data were compared according to the ratio of FPKM and the ratio of mRNA expression level for all selected genes.

### 2.8. Data Analysis

The results were plotted using GraphPad Prism 8.0. Means were compared by least squared difference (LSD) test of one-way analysis of variance (ANOVA) using PROC GLM of SAS program and discriminated at Type I error = 0.05.

## 3. Results

### 3.1. Sequencing, RNA-Seq Assembly, and Functional Annotation

Illumina raw data was qualified by filtering to reveal the transcriptome responses to cold stress in *S. japonica* (Supplementary Tables S3 and S4). In total, 25.1 Gb of clean data passed the Illumina quality filter after transcriptome sequencing of four cDNA samples with Q30 > 95% (Supplementary Table S4). To perform the de novo transcriptome assembly, all high-quality reads (Supplementary Table S4) were pooled. Using paired-end joining and clustering according to the similarity of contigs, these contigs were further assembled into 118,655 transcripts with a mean length of 876.07 bp and an N50 of 1853 bp, and 89,763 unigenes with a mean length of 676.49 bp and an N50 of 1194 bp (Supplementary Tables S5 and S6). The length distribution of unigenes closely followed the length distribution of transcripts. This indicates a high-quality assembly, providing a sequence basis for future studies.

### 3.2. Annotation of Predicted Proteins

The assembled unigenes were blasted against ten public databases (NR, NT, UniProt, Pfam, GO, EggNOG, KEGG, KO_EUK_Annotation, KO_PRO_Annotation, KO_BAC_Annotation and KO_BAC.NUC_Annotation) using BLASTX with a cut off *E*-value of 10–5 for validation and annotation. After annotation, genes with a significant blast hit to arthropods were identified. At most, 32,782 (35.56%) unigenes were found in the NR public database, followed by the NT database (29,598 annotated unigenes, 33.01%), and the KO_EUK_Annotation database (29,144, 32.51%) (Table 1). Overall, most of the unigenes either could not be annotated or their descriptions were uninformative (e.g., putative, unknown, hypothetical, or unnamed proteins). Overall, the unigene sequences were most similar to gene sequences from *Solenopsis invicta* (33.97%) and totally more than 58% similarity with ant genus (*Solenopsis* sp., *Trachymyrmex* sp., *Acromyrmex* sp., *Atta* sp., *Camponotus* sp., *Cyphomyrmex* sp., *Monomorium* sp., *Vollenhovia* sp., *Wasmannia* sp., *Harpegnathos* sp., and *Lasius* sp.) and, interestingly, 12.26% similarity with *Rhagoletis zephyria* (Diptera: Tephritidae) and 11.3% similarity with two genus of mites including *Sacroptes* sp. and *Euroglyphus* sp. were observed via BLASTX matches in the NR database.

ORF prediction for unigenes was performed using the TransDecoder program. ORFs with at least 100 amino acids long were extracted. In total, 21.62% (19,384) of the total predicted unigenes (89,657) were included in at least one ORF (Table 2). From 22,442 predicted ORF, 9904 (44.13%) showed as a full open reading frame (Table 2).

### 3.3. GO and EggNOG Analysis for Global Functional Classification

**Figure 1:**
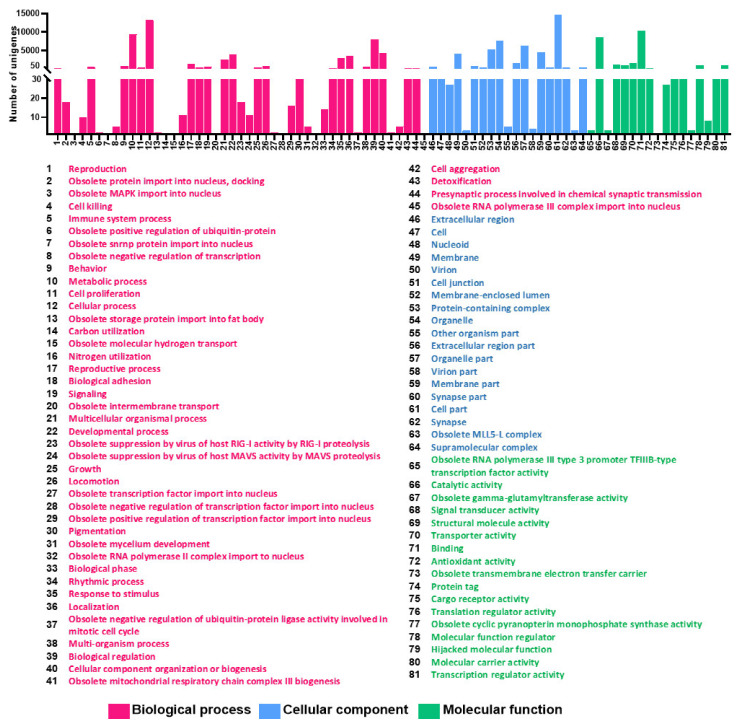
Annotations of the annotated unigenes. The Gene Ontology (GO) classification of all annotated unigenes. A total of 20,645 unigenes were assigned to three main GO categories of ‘Biological process with 45 functional subcategories’, ‘Cellular component with 19 functional subcategories’ and ‘Molecular function with 17 functional subcategories’. The *Y*-axis shows the number of genes in each category. The numbers on the X-axis in each figure correspond to functional subcategories for which a definition is provided.

For functional annotation of the unigenes, the GO database and EggNOG database were applied to classify the annotated unigenes using BLASTX. As one unigene can have different functional annotations, a total of 20,645 genes have been annotated with GO terms inferred from BLAST results. Eighty-one GO functional sub-groups could be obtained according to the three main GO groups ‘biological process (45)’, ‘cell component (19)’ and ‘molecular function (17)’ in the GO database. A total of 8004 unigenes belonged to the biological process group, 6266 unigenes fell into the cellular component group, and 6375 unigenes were categorized in the molecular function group (Figure 1). The most common DEGs coregulated under cold stress according to GO categorization were ‘cellular process’, ‘biological regulation’, ‘metabolic process’, ‘developmental process’, ‘cellular component organization or biogenesis’ and ‘localization’ in the biological process category. In the molecular function category, the coregulated DEGs were mostly assigned to ‘catalytic activity’, ‘binding’, and ‘transporter activity’. For the cellular components category, only ‘membrane’, ‘protein-containing complex’, ‘organelle’, ‘organelle part’, ‘cell part’, and ‘membrane part’ were significantly enriched (Table 3).

**Figure 2:**
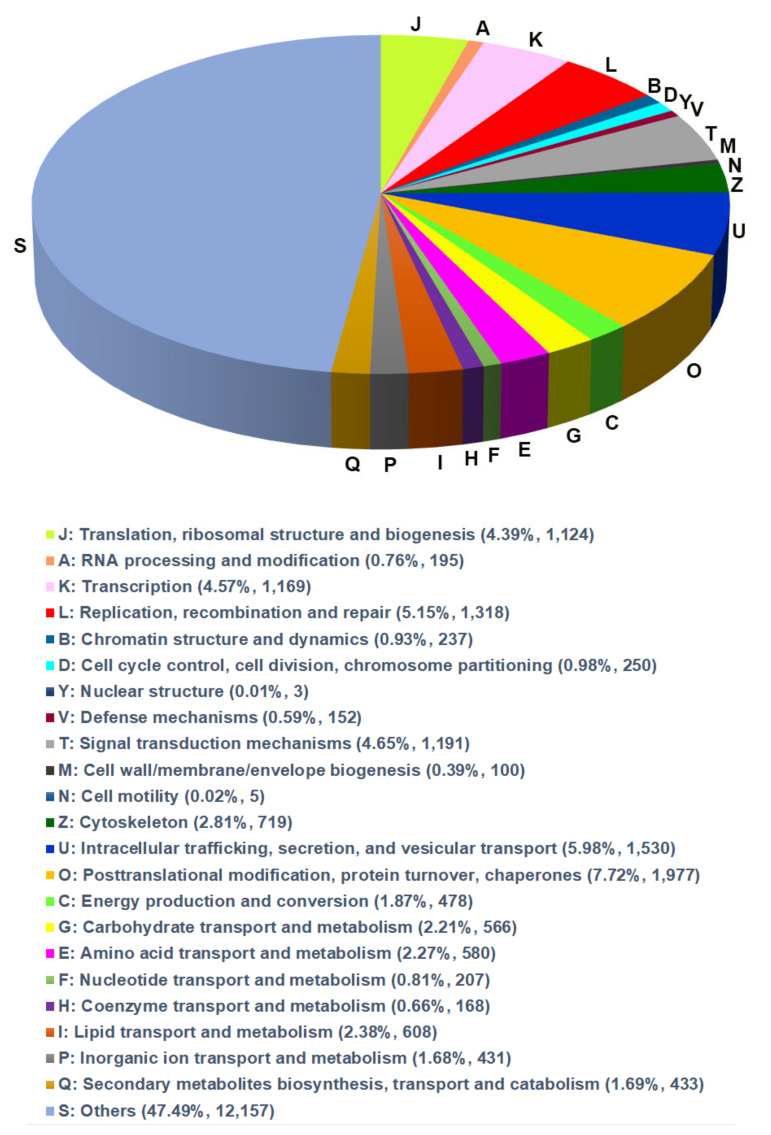
Annotations of the annotated unigenes. The annotated unigenes are mapped to the annotation of the corresponding orthologous groups in EggNOG (evolutionary genealogy of genes) database.

To reveal functional and biological classification, the EggNOG database was used. In total, 26,823 unigenes were assigned to 18 EggNOG terms (Figure 2) that belonged to three functional classes including ‘information storage and processing’, ‘cellular processes and signaling’ and ‘metabolism’. The largest number of unigenes were classified at ‘translation, ribosomal structure and biogenesis (1124 unigenes)’, ‘transcription (1169)’, ‘replication, recombination and repair (1318)’, ‘intracellular trafficking, secretion, and vesicular transport (1530)’ and ‘post-translational modification, protein turnover, chaperones (1977)’ (Figure 2).

### 3.4. Differentially Gene Expression under Different Temperature

**Figure 3:**
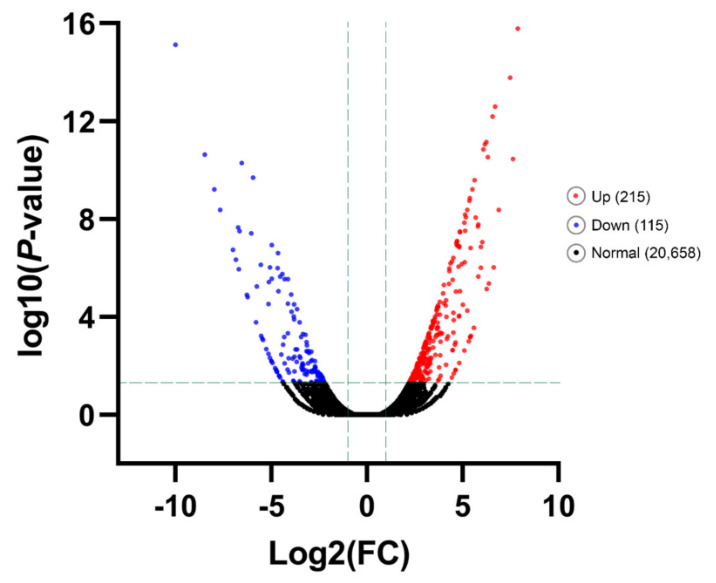
Significant Differentially Expressed Gene (DEG) analysis. The number of all up- and down-regulated contigs based on *p*-value < 0.05 and log_2_FC ≥ 1 or log_2_FC ≤ −1 of comparison pairs was plotted. Volcano diagram for distribution of the identified DEGs in treatment group T9 in comparison with T25 as control was plotted. Red and blue points represent the significant DEGs with *p*-value < 0.05 and log_2_FC ≥ 1 or log_2_FC ≤ −1 and black ones show those without significant DEGs, respectively.

DEGs under cold-stress temperature treatment (T9) compared with the control (T25), were identified. An amount of 330 unigenes were DEGs for T9, with a criterion of adjusted *p*-value < 0.05 and |log2FC| ≥ 1 that 215 unigenes were up-regulated and 115 DEGs down-regulated (Figure 3, Supplementary Tables S7 and S8).

**Figure 4:**
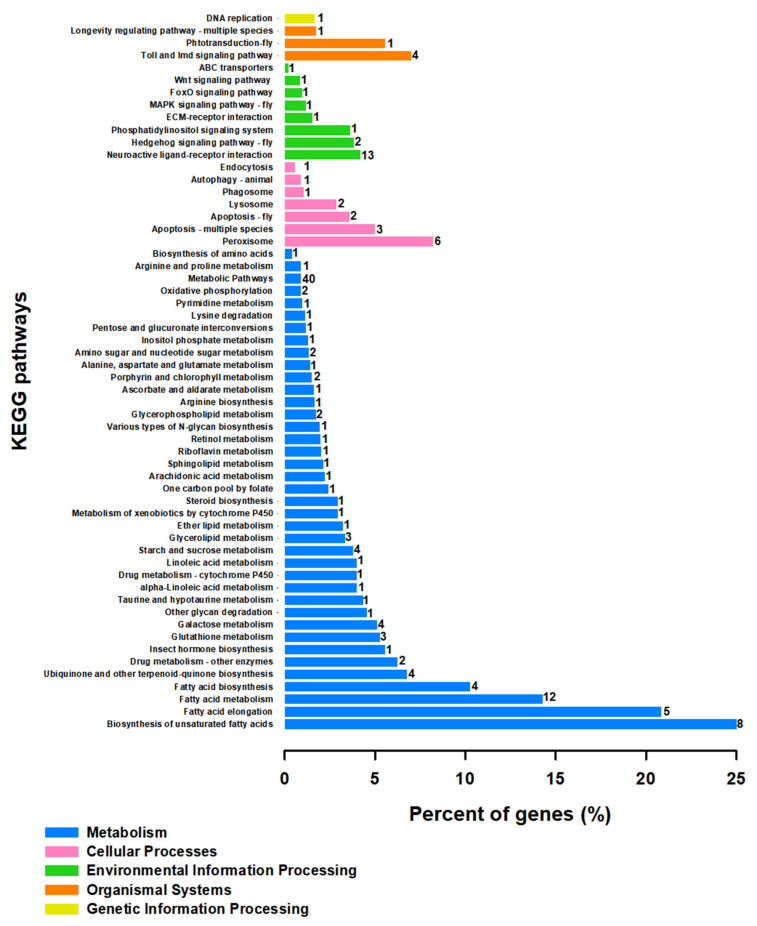
Analysis of significant KEGG pathways enrichment in the specific differentially expressed genes (DEGs) (*p*-value < 0.05, log_2_FC ≥ 1, ≤ −1) coregulated by cold temperature stresses. The Y-axis indicates the KEGG pathways, and the *X*-axis indicates the percentage of unigenes in each pathway, indicated on the left side of bars. The numbers on the right side of bars show the numbers of DEGs significantly enriched in the relevant pathways.

**Figure 5:**
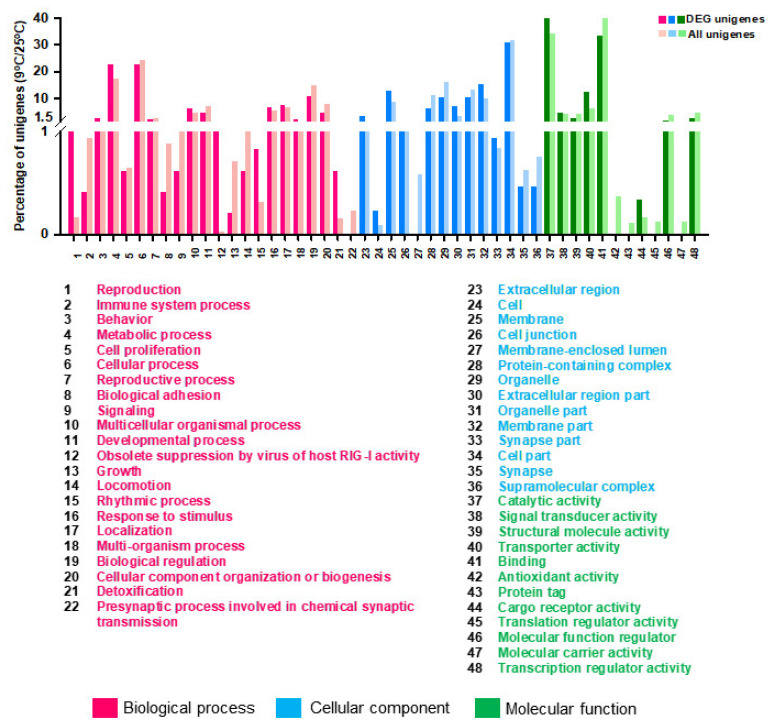
Gene Ontology (GO) annotations of the specific Differentially Expressed Genes (DEGs) (*p*-value < 0.05, log_2_FC ≥ 1, ≤ −1) coregulated by cold temperature stresses in comparison to all DEGs. Go terms are summarized in three main categories of ‘Biological process’, ‘Cellular component’ and ‘Molecular function’. The Y-axis shows the percentage of unigenes in each category.

To clear functional classification, KEGG and GO analysis were performed on screened unigenes (Figure 4, Supplementary Table S9 and Figure 5, Supplementary Table S10, respectively). KEGG pathway enrichment analysis revealed the primary DEG pathways (Figure 4, Supplementary Table S9). A large number of coregulated DEGs under cold temperature stress were significantly enriched in the ‘Metabolic pathways’ which are dominated by ‘α-glucosidase-like’, ‘Uridine 5′-monophosphate synthase-like’, ‘Cysteine sulfinic acid decarboxylase’, ‘Inositol-triphosphate 3-kinase A’, ‘4-coumarate-CoA ligase 1-like’, ‘Pancreatic triacylglycerol lipase-like’, ‘Lipase member H-B like’ and ‘NADH dehydrogenase subunits’ unigenes. Another identified enriched-pathway for the T9 group included ‘Fatty acid metabolism’, which includes ‘Elongation of very long chain fatty acids protein 7-like’, ‘Fatty acid synthase-like’ and ‘Acyl-CoA Delta (11) desaturase’ unigenes. ‘Biosynthesis of unsaturated fatty acids’, ‘Fatty acid elongation’ and ‘Fatty acid biosynthesis’ are other pathways enriched under the effects of low temperature. The protein present in the ‘Peroxisome’-enriched pathway was ‘Fatty acid CoA-reductase’. ‘Trypsin-1-like’, ‘Chymotrypsin-1 and 2-like’, ‘Transmembrane protease serine 9-like’, ‘γ-aminobutyric acid receptor α-like’, ‘Chymotrypsin-like protease CTRL-1′ and ‘Anionic trypsin-2-like’ were the changed participants of the ‘Neuroactive ligand–receptor interaction’ under cold stress conditions. A series of unigenes related to the immune system of ‘Peptidoglycan-recognition proteins’ were responsible for enriching the ‘Toll and Imd signaling pathway’. ‘Glycerolipid metabolism’, Lysosome’, ‘Glycerophospholipid metabolism’, ‘Starch and sucrose metabolism’, and ‘Galactase metabolism’ are other pathways that are enriched at lower temperatures in comparison with 25 °C (Figure 4, Supplementary Table S9).

GO annotation was used to clarify the functions of DEGs that were significantly different (FC ≥ 2 or ≤ −2) with incubation of *S. japonica* at 9 °C in comparison with 25 °C (Figure 5, Supplementary Table S10). The most significant GO items were ‘Biological process: metabolic process, multicellular organismal process, response to stimulus and localization’, ‘Cellular component: membrane, membrane part and extracellular region part’ and ‘Molecular function: catalytic activity and transporter activity (Figure 5, Supplementary Table S10).

### 3.5. Validation of Gene Expression Profiles by qPCR

**Figure 6:**
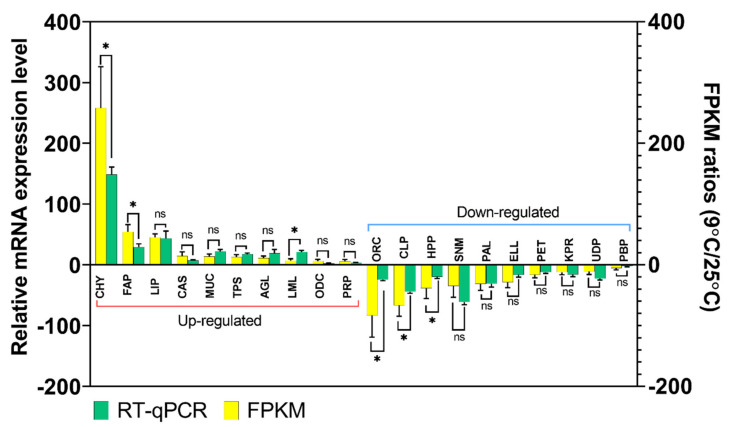
Differentially Expressed Genes (DEGs) validation by RT-qPCR in comparison to corresponding folding change (FC) data detected in RNA-Seq. Relative genes expression (T9/T25) is shown as the ratio of them per *rpl32.* Each treatment includes three technical replicates. Asterisks above standard deviation bars indicate significant difference between groups at Type I error = 0.05 (LSD test). The letter ‘ns’ stands for a non-significant difference between groups.

qPCR and gel electrophoresis of twenty common DEGs identified in the RNA sequence data were performed to confirm the accuracy and reproducibility of the Illumina RNA-Seq. The ten up-regulated DEGs included chymotrypsin-2-like (CHY; c17281_g1_i1), elongation of very long-chain fatty acids, protein 7-like (FAP; c113360_g1_i1), lipase 3-like (LIP; c89947_g1_i2), caspase-1-like (CAS; c90816_g2_i2), mucin 17-like (MUC; c61818_g1_i1), transmembrane protease serine 9-like (TPS; c88786_g3_i1), α-glucosidase-like (AGL; c80199_g1_i1), luciferin 4-monooxygenase-like (LML; c86095_g2_i2), ornithine decarboxylase 2-like (ODC; c77768_g1_i1) and peptidoglycan-recognition protein 1-like (PRP; c78499_g1_i2). Another 10 DEGs that showed down-regulation at T9 compared to T25 were validated by qPCR, including: odorant receptor coreceptor (ORC; c89345_g3_i1), chitinase-3-like protein 1 (CLP; c82068_g1_i1), homeobox protein prophet of Pit-1 (HPP; c78623_g1_i1), sensory neuron membrane protein 1-like (SNM; c90181_g1_i1), phospholipase A1-like (PAL; c79106_g1), elastin-like (ELL; c83852_g1_i2), peritrophin 48-like (PET; c85316_g3_i2), keratinocyte proline-rich protein-like (KPR; c26648_g1_i1), UDP-glucuronosyltransferase 2B18-like (UDP; c69128_g2_i2) and paired box protein Pax 6-like (PBP; c86999_g1_i1). The results of the qPCR and Illumina FPKM ratio were plotted in Figure 6. It can be revealed that expression changes were in the same direction as the qPCR. The Illumina sequencing data were consistent with qPCR data, verifying the reliability and accuracy of the transcriptome analysis. This ensures the RNA-Seq results was considerably reliable for the identification of DEGs under stressed conditions in this study and also the feasibility and sustainability of our further research on these or other DEGs from the transcriptome data.

## 4. Discussion

Insects show different responses to temperature changes as well as distribution and development among animals, which is a very vital factor for predators (Ran et al., 2020). *S. japonica* is a soil dwelling invertebrate predator. Unfortunately, there is a poor understanding of its biology. Comprehensive investigation of gene expression regulation under cold stress is very important to understand the biochemical and physiological adaptation process [22]. The present study tries to show how *S. japonica* can adapt to the low temperature environment for its survival and breeding. Transcriptome sequencing data indicated 330 transcripts of *S. japonica* that were significantly differentially expressed under low temperature stress. This data explains that *S. japonica* has adaptability to low temperatures and can survive under cold stress conditions. Many physiological and biochemical strategies help insects deal with stress conditions involving low temperatures [45]. KEGG analysis revealed that most of the cold-regulated DEGs were enriched in the ‘Metabolism’ pathways, including ‘metabolic pathway’, ‘fatty acid metabolism’, ‘biosynthesis of unsaturated fatty acids’, ‘fatty acid elongation’, ‘fatty acid biosynthesis’, ‘galactase metabolism’ and ‘starch and sucrose metabolism’. There are some similar results in the investigation into transcriptome responses to cold stress in the chrysomelid, *Galeruca daurica* [22], the ladybird, *Cryptolaemus montrouzieri* [21,22], and also the desert beetle, *Microdera punctipennis* [46].

In this study, we identified 15 serine protease protein genes from 215 up-regulated DEGs after cold stress (Supplementary Table S7), and also two chymotrypsin genes down-regulated (Supplementary Table S8). The accumulation of serine protease, predominantly chymotrypsin mRNA, indicates that adults are preparing for diet feeding and subsequent diapausing because, along with serine proteases, 12 fatty acid synthase and fatty acyl-CoA reductase continue to be expressed. This pattern is evident that regulation of fatty acid synthase and serine proteases is a part of the diapause program and not a result of other factors such as temperature, host availability, or feeding activities [47]. However, there are some reports of fatty acid accumulation in insects that were incubated under high-temperature stress [45,48]. One opinion is that fatty acids are hydrophobic, allowing insects to keep body water in hostile environments.

The KEGG results revealed that carbohydrate metabolic processes, including ‘galactase metabolism’ and ‘starch and sucrose metabolism’ are the other main pathways and three α-glucosidae, as an important group of enzymes that are specialized in sugar digestion [49], are up-regulated when *S. japonica* is exposed to low temperatures, indicating *S. japonica* mainly metabolized sugar during low-temperature stress. In addition, several amino acids were detected and identified in cold stress conditions, including taurine, arginine, proline, alanine, aspartate, and glutamate (Figure 4), which have been previously reported [50,51,52,53]. Under the cold-stress condition, ants showed enriched pathways of both proline and arginine than room temperature-exposed ants, and contained an enriched pathway of glutamate, a precursor to arginine and proline (Figure 4, Supplementary Table S9). Together, these findings suggest that these polar and charged amino acids, and modulation of proline metabolism in particular, may be essential to the achievement of cold tolerance in *S. japonica*, as the same condition was observed for *Drosophila melanogaster* [50]. Glutamate is not only a metabolic precursor of arginine and proline, but also of glutathione [50], and ‘Glutathione metabolism’ was one of the recorded KEGG-enriched pathways in the ants that was exposed at low temperature in our findings (Figure 4, Supplementary Table S9). Cold acclimation also involved significant up-regulation of genes involved in immunity. Protein spaetzle, peptidoglycan-recognition protein, Toll-like receptor and transferrin-like peptide are associated with cold tolerance in *S. japonica*. A relationship between cold exposure and immunity has also been suggested by numerous transcriptomic studies [54,55,56], although the nature of this relationship remains unclear [50]. Peritrophin genes related to the peritrophic membrane, which have been previously suggested, appear to be of great importance to insect cold tolerance [57] and are up-regulated in our insect model *S. japonica* when incubated at a low temperature (Supplementary Table S7).

In this research, we also discovered 12 DEGs related to odorant receptor proteins (ORPs) were down-regulated (Supplementary Table S8). This shows that the chemosensory system of *S. japonica* probably cannot respond properly to chemical cues in the environment at low temperatures. Microarray transcriptomic studies of the third antennal segment of high-temperature-acclimated *Drosophila* revealed that four ORPs were up-regulated and five of them down-regulated. Indeed, low temperature and some stress conditions such as starvation have been connected to alteration of some ORPs [58]. Under cold treatment, several enzymes, including NADH dehydrogenase subunit and ATP synthase subunit that are involved in ‘oxidation phosphorylation’ pathway, were significantly down-regulated. This was consistent with findings of cold tolerance of a lepidopteran insect, *Eogystia hippophaecolus* [43]. The CYP family is also a superfamily associated with oxidative metabolism. It has been previously reported that CYP is related to temperature regulation [44,59]. In the present study, several genes encoding CYP (10 mRNAs) were also found to be significantly up-regulated under cold stress. However, two genes related to CYP were down-regulated at low temperatures.

HSP, as a highly conserved molecular chaperone, plays an important role in organism responses to stressor conditions, such as cold hardiness, that can have an effect on the folding and functional conformation of proteins [28,60]. Compared with previous studies that proved HSP genes can be induced to express by cold and heat stress [44,60], we could not find many HSPs. We found just two HSPs (Table S8) that were down-regulated by cold stress at 9 °C for 24 h. There is a report that many HSP genes are differentially expressed under the cold (9 °C for 24 h) in ladybirds [21]. Tusong et al. 2016, also discovered seven HSPs (HSP90 and HSP70) up-regulated by cold stress in a coleopteran insect, *Metapogon punctipennis* [46]. This difference might be due to the differences in HSPs, insect species, and intensity of temperature treatment. As reported, sex intensity of treatment and insect strain can be affected by HSP induction [22,35].

## 5. Conclusions

The recent analysis discovered many DEGs, but their roles at low temperatures are unknown. However, determining the molecular pathways underlying a complicated process purely on the basis of the involvement of these DEGs is difficult, but it will be fascinating if the significance of these DEGs is elucidated in the future. In conclusion, we present a global survey of the transcriptome profile in *S. japonica* under cold stress condition using RNA-Seq technology. Comparative transcriptome study showed numerous genes and pathways that are ubiquitous under cold stress, leading to speculation on the molecular basis of this fire ant. This is the first study of cold stress induction in *S. japonica* and we first identified genes in this study, including some functionally unknown genes. These newly found genes may be important specifically in the cold environment. Our data will facilitate further molecular investigations into the cold tolerance of *S. japonica* and provide new insights into insect adaption to the harsh environment.

## Figures and Tables

**Table 1:** Statistics of annotation analysis of unigenes.

Database	Unigene	300 ≤ Length < 1000	Length ≥ 1000
NT_Annotation	29,598 (33.01%)	11,806	11,029
NR_Annotation	32,782 (36.56%)	12,648	10,156
Pfam_Annotation	20,267 (22.61%)	6890	8242
EggNOG_Annotation	26,823 (29.92%)	9520	9796
KO_EUK_Annotation	29,144 (32.51%)	10,924	9738
KO_PRO_Annotation	6889 (7.68%)	2126	3564
KO_BAC_Annotation	6376 (7.11%)	1877	3469
KO_BAC.NUC_Annotation	242 (0.27%)	79	115
GO_Annotation	20,645 (23.03%)	6775	8254
UniProt_Annotation	19,121 (21.33%)	6182	7782

**Table 2:** Statistics of Open Reading Frame (ORF) prediction.

Assembly	Total Unigene		ORF Predicted Unigene	Single ORF Predicted Unigene	Multiple ORF Predicted Unigene
Merge	89,657		19,384 (21.62%)	16,956 (87.47%)	2428 (12.53%)
**Assembly**	**No. of ORF**	**Complete**	**Internal**	**5′ partial**	**3′ partial**
Merge	22,442	9904 (44.13%)	6116 (27,25%)	4790 (21.34%)	1632 (7.27%)

**Table 3:** Significantly enriched gene ontology (GO) terms in the differentially expressed genes (DEGs) coregulated by cold stresses.

GO Term	Unigenes	DEGs (FC ≥ 2, FC ≤ −2)	Corrected *p*-Value
**Biological Process**			
Cellular process	13,159 (24.32%)	109 (22.52%)	0.003627
Metabolic process	9319 (17.22%)	110 (22.73%)	4.8 × 10^−5^
Biological regulation	8026 (14.83%)	52 (10.74%)	0.004567
Developmental process	3838 (7.09%)	23 (4.75%)	0.002454
Cellular component	3971 (7.45%)	65 (1.63%)	2.85 × 10^−5^
Localization	3515 (6.5%)	36 (7.44%)	0.000132
**Cellular Component**			
Cell part	14,694 (31.76%)	132 (30.91%)	0.000105
Organelle	7513 (16.24%)	45 (10.54%)	0.00182
Membrane part	4536 (9.8%)	65 (15.22%)	0.036674
Protein-containing complex	5210 (11.26%)	26 (6.09%)	0.000455
Membrane	3978 (8.6%)	55 (12.88%)	0.004567
Organelle part	6190 (13.38%)	45 (10.54%)	1.02 × 10^−9^
**Molecular Function**			
Binding	10,355 (41.73%)	97 (33.22%)	3.29 × 10^−14^
Catalytic activity	8531 (34.38%)	124 (42.47%)	0.00136
Transporter activity	1565 (6.31%)	36 (12.33%)	5.71 × 10^−6^

## Data Availability

NGS data of *Solenopsis japonica* used in this study has been deposited in the Gene Expression Omnibus (GEO) database (https://www.ncbi.nlm.nih.gov/geo/, accession No, GSE155469) and the Sequence Read Archive (SRA) (https://www.ncbi.nlm.nih.gov/sra, accession No. SRP274474).

## Supplementary Materials

The following are available online at https://www.mdpi.com/article/10.3390/genes12101610/s1, Table S1: Primer sequences used in this study. Table S2: Project information of transcriptome analysis. Table S3: Summary of data production (Raw data statistics results). Table S4: Summary of data production (Trimming data statistics results). Table S5: De novo transcriptome assembly statistics. Table S6: Clustering transcripts into unigenes. Table S7: Specific and core T9 associated DEGs in compare with T25 that up-regulated (*p*-value < 0.05, log2FC ≥ 1). Table S8: Specific and core T9 associated DEGs in compare with T25 that down-regulated (*p*-value < 0.05, log2FC ≤ −1). Tabls S9: Associated genes related to pathways enrichment base on KEGG classification (*p*-value < 0.05, log2FC ≥ 1, ≤ −1). Table S10: Significantly enriched GO terms in the DEGs coregulated by cold stresses, 9℃, (T9) in compare with T25, (*p*-value<0.05, log2FC ≥ 1, ≤ −1).
