# Supplementary material for: Comparative RNA-Seq Analyses of Solenopsis japonica (Hymenoptera: Formicidae) Reveal Gene in Response to Cold Stress
Source: Genes (Basel). 2021 Oct 13;12(10):1610. doi: 10.3390/genes12101610 (PMC8535336; doi:10.3390/genes12101610)
Supplement: Supplementary file 1 [file genes-12-01610-s001.zip › genes-1336859-supplementary.pdf]

GENES

Comparative RNA-Seq analyses of *Solenopsis japonica* (Hymenoptera: Formicidae) reveal gene in response to cold stress

Mohammad Vatanparast, Youngjin Park*

Plant Quarantine Technology center, Animal and Plant Quarantine Agency, Gimcheon, Korea

*Corresponding author

Email) parky1127@korea.kr

Supplementary data

Table S1. Primer sequences used in this study.

Table S2. Project information of transcriptome analysis.

Table S3. Summary of data production. Raw data statistics results. Q20 and Q30 are Ratio (%) of bases that have phred quality score greater than or equal to 20 or 30, respectively.

Table S4. Summary of data production. Trimming data statistics results. Adapter sequences were removed. Q20 and Q30 are Ratio (%) of bases that have phred quality score greater than or equal to 20 or 30, respectively.

Table S5. *De novo* transcriptome assembly statistics. The *de novo* assembly of merged data was carried out using Trinity software. N[x] length statistic: At least x % of the assembled transcript nucleotides are found in contigs that are at least of Nx length.

Table S6. Clustering transcripts into unigenes. Longest contigs of the assembled contigs are filtered and clustered into the non-redundant transcripts using CD-HIT-EST program. N[x] length statistic: At least x % of the assembled transcript nucleotides are found in contigs that are at least of Nx length.

Table S7. Specific and core T9 associated DEGs in compare with T25 that up-regulated (*P*-value<0.05, $log_2FC \geq 1$).

Table S8. Specific and core T9 associated DEGs in compare with T25 that down-regulated (*P*-value<0.05, $log_2FC \leq -1$).

Table S9. Associated genes related to pathways enrichment base on KEGG classification (*P*-value<0.05, $log_2FC \geq 1$, ≤-1).

Table S10. Significantly enriched GO terms in the DEGs coregulated by cold stresses, 9℃, (T9) in compare with T25, (*P*-value<0.05, $log_2FC \geq 1$, ≤-1).

Table S1.

Primer for unigenes	Abbreviation	Use	Orientation	Sequence (5' - 3')	Annealing temperature (°C)	Amplicon (bp)
Sj_chymotrypsin	CHY	qPCR	Forward<br>Reverse	GGAGCAAGTTTGTGCGTATAAC<br>GCCAATCCGACGAGTTTCT	52	99
Sj-fatty acids protein	FAP	qPCR	Forward<br>Reverse	AAGCCTCGGTCCAAGATTAAATA<br>GGCAGATCAACGTACCAAAGA	52	106
Sj-lipase 3-like	LIP	qPCR	Forward<br>Reverse	TTGGCAACACACGAGGTAATA<br>CATGGCAGGAAGGTCGTAAA	52	116
Sj-caspase-1-like	CAS	qPCR	Forward<br>Reverse	CGCGAAGGACGACAAATACAGA<br>TTGCAAGCCTGGATGAAGAATAG	52	107
Sj-mucin-17-like	MUC	qPCR	Forward<br>Reverse	CCGTTCTCCAGGACGATAAAC<br>GAGACGACCCATGAAAGTAACA	52	109
Sj-transmembrane protease	TPS	qPCR	Forward<br>Reverse	GATCAGGACGTTAAGACCAATGA<br>ATGAGGATGTTGACTCGTTGTAG	52	104
Sj-alpha-glucosidase	AGL	qPCR	Forward<br>Reverse	GTGGAGGGCTTTCGTATTGA<br>CGTAGTCATCCGGTGGTAAAG	52	100
Sj-luciferin 4-monooxygenase	LML	qPCR	Forward<br>Reverse	ACTACTCGCATTGTCATCTTCC<br>CGGGCACGTTTGCTTATTTAC	52	130
Sj-ornithine decarboxylase	ODC	qPCR	Forward<br>Reverse	CAAGCCGACCGGATCATATT<br>TTCGCTGTCGGCAGTTATC	52	99
Sj-peptidoglycan-recognition protein	PRP	qPCR	Forward<br>Reverse	CCGGGTAGGGTAACTACATACT<br>GCAAGATAAGGGACAGCTACAA	52	100
Sj-odorant receptor	ORC	qPCR	Forward<br>Reverse	GATCTAAGGACACCGTGAAGAA<br>GATGGATCGGAGGAAGCTAAA	52	103
Sj-chitinase	CLP	qPCR	Forward<br>Reverse	TCGTTGTATCCGAGCATTCC<br>AGCCTTCACTCTTGCTGATAAA	52	108
Sj-homeobox protein	HPP	qPCR	Forward<br>Reverse	GGACGAGTGGTACAACAAGAG<br>CTCTCGCCTCCGTTTACAATTA	52	113
Sj-sensory neuron membrane	SNM	qPCR	Forward<br>Reverse	CGGCCCACGTCTTCATTATT<br>CGAGAGGATGGTTTACGGTTTAG	52	113
Sj-phospholipase A1	PAL	qPCR	Forward<br>Reverse	TTGCCACGAGTTCACCTATC<br>GCGAGACGGCACTTGTAATA	52	123
Sj-elastin	ELL	qPCR	Forward<br>Reverse	GTGTTAGCGGCTGAGAAGAA<br>CCGTAGGTCGAGATACCATCTA	52	104
Sj-peritrophin	PET	qPCR	Forward<br>Reverse	GCGATTGCTCAAGGAATTGTATC<br>CAACCGTGTTCAACCTTCTTTC	52	97
Sj-keratinocyte	KPR	qPCR	Forward<br>Reverse	CCCTTAACGACCACTGGATATG<br>CAAGGTCCCGGTCGAAATAG	52	90
Sj-glucuronosyltransferase	UDP	qPCR	Forward<br>Reverse	AGCATCCGGAAGTACGTAAGA<br>CTATGCGCCAAGGCATACAA	52	106
Sj-paired box protein	PBP	qPCR	Forward<br>Reverse	GAGGAGTTGTGCTCCGAATTAC<br>CGGGCGTGACGATTCTTTAT	52	99
Sj_rpl32	*rpl32*	qPCR	Forward<br>Reverse	GCAACTGGCGTAAACCAAAG<br>CTTGCGGAAACCAGTAGGTAG	52	131

Table S2.

**Project information**	
Read Length	101
Library Kit	TruSeq Stranded mRNA LT Sample Prep Kit
Library Protocol	TruSeq Stranded mRNA Sample Preparation Guide, Part # 15031047 Rev.
Reagent	TruSeq 3000 4000 SBS Kit v3
Sequencing Protocol	HiSeq 3000 4000 System User Guide Document # 15066496 v05 HCS 3.3.52
Sequencing Control Software	HCS 3.3.52

Table S3.

Index	Sample ID	Total read bases	Total reads	Throughput (Gb)	GC (%)	Q20 (%)	Q30 (%)
1	T25	13,180,831,280	130,503,280	13.25	40.03	97.35	93.51
2	T9	12,894,845,134	127,671,734	12.9	43.36	98.41	95.51

Table S4.

Index	Sample ID	Total read bases	Total reads	Throughput (Gb)	GC (%)	Q20 (%)	Q30 (%)
1	T25	12,543,606,644	126,549,750	12.6	40.14	98.53	95.4
2	T9	12,499,689,974	124,945,436	12.5	43.37	99.05	96.63

Table S5.

Assembly	Merge	
	All transcript contigs	Only longest isoform per'gene'
Total trinity 'genes'	89,763	89,763
Total trinity transcripts	118,655	89,763
Percent GC	41.71	41.74
N90	302	258
N80	492	349
N70	811	498
N60	1,290	748
N50	1,853	1,194
N40	2,477	1,831
N30	3,204	2,631
N20	4,217	3,635
N10	5,720	5,219
Maximum contig length	16,238	16,238
Minimum contig length	201	201
Median contig length	390.0	334.0
Average contig length	876.07	676.49
Total assembled bases	103,950,075	60,723,397

Table S6.

Assembly	Merge	
	Only longest isoform per'gene'	Clustered Contig 'Unigene'
Total 'genes'	89,763	89,657
Percent GC	41.74	41.74
N90	258	258
N80	349	349
N70	498	498
N60	748	748
N50	1,194	1,195
N40	1,831	1,832
N30	2,631	2,633
N20	3,635	3,638
N10	5,219	5,222
Maximum contig length	16,238	16,238
Minimum contig length	201	201
Median contig length	334.0	334.0
Average contig length	676.49	676.76
Total assembled bases	60,723,397	60,676,578

Table S7.

No	Contig	Gene	Accession number	Fold change	FPKM	
					T9	T25
1	c17281_g1_i1	Chymotrypsin-2-like	LOC105200925	235.9	13033	51
2	c88644_g1_i1	Facilitated trehalose transporter Tret1-like	LOC105198897	198.65	455	2
3	c90698_g1_i1	Genome polyprotein	GO:0039520	178.76	3307	17
4	c78533_g1_i1	Genome polyprotein	GO:0039546	118.34	271	2
5	c88505_g1_i1	Salivary secreted peptide	LOC105196694	103.82	42533	379
6	c69835_g1_i1	Retinoid-inducible serine carboxypeptidase-like	LOC105200222	98.41	119	1
7	c102119_g1_i1	Chymotrypsin-1-like	LOC108727998	94.95	24637	240
8	c75529_g1_i1	Maltase 1-like	LOC105828798	82.72	100	1
9	c90583_g2_i3	Reverse ribonuclease integrase	LOC105205348	79.39	1726	20
10	c107777_g1_i1	Rho gtpase-activating protein	GO:0005096	76.94	93	1
11	c88505_g2_i1	Salivary secreted peptide	LOC105196696	75.54	16092	197
12	c106856_g1_i1	Protein G12-like	LOC105201876	71.89	38543	496
13	c85916_g1_i1	Salivary secreted peptide	LOC105196697	68.28	29448	399
14	c70911_g1_i2	Protein amnionless-like	LOC105197205	65.29	220	3
15	c74904_g1_i2	Vitellogenin-3	LOC105205783	62.03	142	2
16	c81173_g1_i1	Genome polyprotein	GO:0039544	61.73	208	3
17	c73740_g2_i1	Alpha-glucosidase-like	GO:0003824	56.42	373	6
18	c85360_g2_i1	Monocarboxylate transporter 9	LOC105193726	56.36	129	2
19	c65440_g1_i3	Histone-lysine N-methyltransferase SETMAR-like	LOC105201911	55.36	366	6
20	c89980_g2_i1	Fatty acyl-coa reductase 1	LOC105200177	51.56	508	9
21	c88786_g3_i1	Transmembrane protease serine 9-like	LOC105200924	49.74	27634	514
22	c80861_g2_i2	Cell wall integrity and stress response component 4-like	LOC105198571	48.02	58	1
23	c67360_g1_i3	Chymotrypsin-1-like	LOC105193271	45.51	38463	782
24	c89457_g1_i1	Replicase polyprotein	APG78588.1	43.07	52	1
25	c90634_g3_i2	Fatty acyl-coa reductase 1-like	LOC105204137	42.96	284	6
26	c87865_g1_i1	Sulfotransferase 4A1-like	LOC105199217	42.24	51	1
27	c89069_g2_i1	Leishmanolysin-like peptidase	LOC105568028	42.24	51	1

28	c80199_g1_i1	Alpha-glucosidase-like	LOC105193841	41.4	94084	2103
29	c90078_g1_i1	Envelope fusion protein	GO:0006508	40.81	15837	359
30	c66350_g1_i1	ATP-dependent DNA helicase Q4	LOC105193681	39.76	48	1
31	c80341_g1_i1	Gamma-aminobutyric acid receptor alpha-like	LOC105194410	38.03	87	2
32	c78777_g1_i3	Protein spaetzle 5	LOC105207756	37.76	6493	159
33	c78799_g1_i1	Transient receptor potential cation channel protein painless-like	LOC105194898	35.87	3803	98
34	c73740_g3_i1	Alpha-glucosidase-like	LOC105193841	35.08	1028	27
35	c106909_g1_i1	Transmembrane protease serine 9-like	LOC105194706	34.82	162898	4329
36	c6255_g2_i1	Peritrophin-1-like	LOC105200309	34.72	267	7
37	c112792_g1_i1	Chymotrypsin-1-like	LOC105193272	34.28	2894	78
38	c67341_g1_i1	Rrna promoter binding protein	GO:0003690	33.98	41	1
39	c79791_g1_i1	Aquaporin aqpae.a-like	LOC105207509	30.96	305	9
40	c90634_g5_i1	Fatty acyl-coa reductase 1-like	LOC108770070	29.12	161	5
41	c112594_g1_i1	Chymotrypsin-1	LOC105197985	28.94	87724	2805
42	c88786_g2_i2	Chymotrypsin-2-like	LOC105200925	28.93	19076	610
43	c74709_g1_i4	Neuronal acetylcholine receptor subunit alpha-2-like	LOC105203848	28.43	1079	35
44	c88620_g1_i3	Dappudraft	GO:0048869	28.42	1017	33
45	c80784_g2_i1	Serine protease 53-like	LOC105200928	28.21	95	3
46	c44190_g1_i1	Netrin-A-like	LOC105200681	27.73	423	14
47	c79489_g1_i1	Putative serine protease K12H4.7	LOC105196079	26.54	2298	80
48	c84339_g1_i1	Chymotrypsin-2-like	LOC105193273	26.3	10746	378
49	c101808_g1_i1	Zinc carboxypeptidase-like	LOC105196175	26.23	24838	876
50	c35068_g1_i1	Glucosidase KIAA1161	LOC105194525	25.52	114034	4135
51	c76260_g1_i1	Homeobox homolog Ptx1	LOC108760525	24.94	57	2
52	c88504_g1_i1	Uridine 5'-monophosphate synthase-like	LOC105201038	24.89	30	1
53	c114158_g1_i1	Cysteine sulfinic acid decarboxylase	LOC105202384	24.89	30	1
54	c88969_g4_i1	Mrna cap guanine-N7 methyltransferase-like	LOC105207514	24.06	55	2
55	c89980_g1_i1	Fatty acyl-coa reductase 1	LOC105200177	23.05	2120	85
56	c8622_g1_i1	Facilitated trehalose transporter Tret1-like	LOC105460343	22.88	77	3
57	c88341_g3_i1	Dopamine N-acetyltransferase-like	LOC108763120	22.32	437	18
58	c90297_g13_i1	Inositol-trisphosphate 3-kinase A	LOC105199246	22.32	51	2
59	c68751_g1_i1	Cytochrome P450 6k1-like	LOC105199236	21.86	3215	136

60	c85495_g1_i1	Dehydrogenase-like oxidoreductase	LOC105195503	21.59	26	1
61	c107020_g1_i1	Cytochrome b5-like	LOC105202306	19.93	2048	95
62	c90507_g5_i3	UDP-glucuronosyltransferase 2B31-like	LOC105197256	19.74	4439	208
63	c79993_g1_i2	Fatty acid synthase-like	LOC105198161	18.67	83	4
64	c76163_g1_i1	DDB1- and CUL4-associated factor 8-like	LOC105143086	18	80	4
65	c88646_g1_i3	Juvenile hormone-binding protein-like	LOC105203826	17.91	157	8
66	c119320_g1_i1	4-coumarate--coa ligase 1-like	LOC105196063	17.52	40	2
67	c72170_g1_i3	Peritrophin-1-like	LOC105200311	17.4	303	16
68	c89849_g6_i9	Sodium/potassium/calcium exchanger 3-like	LOC105457875	16.31	425	24
69	c88505_g3_i1	Salivary secreted peptide	LOC105196698	16.3	17106	971
70	c81590_g1_i2	Sodium- and chloride-dependent glycine transporter 2-like	LOC105202689	15.9	1291	75
71	c87356_g3_i1	Genome polyprotein	GO:0039544	14.49	127	8
72	c66823_g1_i1	Chymotrypsin-like protease CTRL-1	LOC105206213	14.18	63	4
73	c86744_g1_i1	Putative inorganic phosphate cotransporter	LOC105196548	14.11	383	25
74	c78680_g1_i1	Pancreatic triacylglycerol lipase-like	LOC105199576	14.02	2683	177
75	c97242_g1_i1	Zinc finger protein	KIAA0543	13.68	46	3
76	c93266_g1_i1	Neutral ceramidase-like	LOC108688619	13.59	31	2
77	c113360_g1_i1	Elongation of very long chain fatty acids protein 7-like	LOC105207261	13.23	2675	187
78	c49886_g1_i1	Transmembrane protease serine 9-like	LOC105194706	13.2	244	17
79	c88262_g1_i1	Furin-like protease 1, isoforms 1/1-X/2	LOC108761537	13.15	30	2
80	c90723_g1_i3	Zinc finger MYM-type protein 1-like	LOC105556737	12.93	337	24
81	c77580_g1_i1	POU domain, class 2, transcription factor 2-like	LOC105834934	12.83	265	19
82	c83980_g1_i1	Protein mesh	LOC105196469	12.78	15481	1121
83	c118191_g1_i1	Peritrophin-1-like	LOC105200309	12.77	18220	1320
84	c48208_g1_i1	Peritrophin-1-like	LOC105194610	12.64	5370	393
85	c77768_g1_i1	Ornithine decarboxylase 2-like	LOC105200774	12.59	260	19
86	c80288_g1_i3	Facilitated trehalose transporter Tret1-like	LOC105566020	12.56	1087	80
87	c77048_g1_i1	Peptidoglycan-recognition protein 1-like	LOC105196158	12.43	203	15
88	c39730_g1_i1	Cytochrome P450 4g15	LOC105203423	12.36	79263	5932
89	c86245_g1_i1	Ran-specific gtpase-activating protein-like	LOC105838716	11.96	79	6
90	c87774_g2_i1	Fatty acid synthase-like	LOC108748804	11.89	207	16
91	c89947_g1_i2	Lipase 3-like	LOC105208099	11.83	1868	146

92	c83670_g1_i1	Lipase member H-B-like	LOC105207817	11.67	1376	109
93	c75351_g1_i2	Trypsin-1-like	LOC105200778	11.36	1401	114
94	c64079_g1_i1	Coiled-coil domain-containing protein 108	LOC105200312	11.08	145	12
95	c87142_g1_i10	Zinc finger MYM-type protein 1-like	LOC105202353	10.99	2436	205
96	c88751_g1_i2	Acyl-coa Delta(11) desaturase	LOC105200008	10.95	29022	2453
97	c87008_g1_i2	Cubilin	LOC105202355	10.94	7247	613
98	c86782_g1_i1	Insulin-like growth factor-binding protein complex acid labile subunit	LOC105196347	10.86	1868	159
99	c89910_g1_i9	Phospholipid-transporting atpase IF	LOC105200500	10.39	1416	126
100	c90816_g2_i2	Caspase-1-like	LOC105198123	10.34	1219	109
101	c89124_g1_i1	Mitochondrial sodium/hydrogen exchanger 9B2	LOC105204367	10.27	90	8
102	c80100_g1_i2	Retinoid-inducible serine carboxypeptidase-like	LOC105200221	10.23	3118	282
103	c21111_g1_i1	Fatty acid synthase	LOC105839757	10.16	58760	5351
104	c88709_g1_i1	Aminopeptidase N	LOC105205293	10.03	12122	1118
105	c89408_g2_i2	RING finger protein nhl-1-like	LOC105620815	9.96	55	5
106	c76996_g1_i2	Acyl-coa Delta(11) desaturase-like	LOC105194093	9.89	76	7
107	c85003_g1_i1	Dystrophin, isoforms A/C/F/G/H-like	LOC105566931	9.89	108	10
108	c87858_g1_i1	Homeobox protein Nkx-2.5-like	LOC105202141	9.72	600	57
109	c78160_g1_i2	Ankyrin repeat domain protein	LOC105202067	9.71	673	64
110	c46607_g1_i2	Ejaculatory bulb-specific protein 3	LOC105202765	9.71	127	12
111	c63403_g1_i1	Inorganic phosphate cotransporter	LOC105196548	9.64	199	19
112	c98501_g1_i1	Cytochrome P450 9e2-like	LOC105205219	9.6	302	29
113	c79953_g1_i2	E3 SUMO-protein ligase EGR2-like	LOC105207564	9.56	600	58
114	c31095_g1_i1	Transient receptor potential cation channel protein painless-like	LOC105205706	9.46	42	4
115	c84537_g1_i4	Fatty acid synthase-like	LOC105198903	9.4	123	12
116	c88025_g3_i1	Fatty acyl-coa reductase CG5065	LOC105203556	9.29	212	21
117	c89094_g1_i1	Chymotrypsin-2-like	LOC105200093	9.28	17899	1784
118	c83419_g3_i2	Fatty acid synthase-like	LOC105196297	9.16	100	10
119	c90490_g1_i1	Aminopeptidase N-like	LOC105193936	9.07	256	26
120	c80951_g1_i1	WD repeat-containing protein 87-like	LOC105204631	8.94	59	6
121	c90414_g1_i2	Type B DNA polymerase	LOC108746207	8.88	5876	612
122	c88979_g2_i4	Elongation of very long chain fatty acids protein	LOC105201316	8.76	285	30

123	c32213_g1_i1	Elongation of very long chain fatty acids protein	LOC105829987	8.73	218	23
124	c76863_g1_i2	4-coumarate--coa ligase 1-like	LOC105196063	8.66	132	14
125	c80636_g1_i1	Fatty acid synthase-like	LOC105202560	8.64	85	9
126	c87215_g3_i1	Zinc finger MYM-type protein 1-like	LOC109862208	8.6	159	17
127	c89338_g5_i1	Growth factor receptor-bound protein 14-like	LOC105448265	8.52	47	5
128	c69192_g1_i1	Integrin beta-like protein 1	LOC105193218	8.51	746	81
129	c11645_g1_i1	Anionic trypsin-2-like	LOC105207761	8.49	1873	204
130	c75449_g1_i1	Protein THEM6	LOC105201010	8.45	10981	1202
131	c86184_g1_i1	Putative nuclease HARBI1	LOC108720640	8.39	636	70
132	c63358_g1_i1	Synaptic vesicle glycoprotein 2B-like	LOC105199891	8.34	1884	209
133	c73792_g2_i2	MFS-type transporter SLC18B1-like	LOC105203816	8.19	3957	447
134	c88690_g1_i1	Maf-like protein CTC_02076	LOC106743984	8.17	2279	258
135	c83526_g2_i1	Cytochrome P450 6A1-like	LOC105205662	8.13	5071	577
136	c102676_g1_i1	CD9 antigen	LOC105198889	7.96	3504	407
137	c90402_g1_i1	Ankyrin repeat domain protein	LOC105448935	7.9	257	30
138	c89078_g11_i1	PX domain-containing protein kinase-like protein	LOC105198808	7.86	2828	333
139	c87162_g1_i4	NADPH--cytochrome P450 reductase	LOC105457599	7.82	432	51
140	c88979_g4_i2	Elongation of very long chain fatty acids protein 7-like	LOC105201228	7.73	719	86
141	c89628_g4_i1	Cytochrome P450 4g15-like	LOC105193720	7.7	58448	7025
142	c38221_g1_i1	Coiled-coil domain-containing protein 13-like	LOC105196650	7.65	340	41
143	c54236_g1_i1	Caspase-1-like	LOC105193867	7.59	616	75
144	c84884_g1_i1	Ribonuclease H-like	LOC108748723	7.56	246	30
145	c108287_g1_i1	Peptidoglycan-recognition protein SC2-like	LOC105564513	7.55	1012	124
146	c114515_g1_i1	Cell wall integrity and stress response component 4-like	LOC105198571	7.53	359	44
147	c80784_g1_i1	Chymotrypsin-2-like	LOC108727746	7.49	195	24
148	c71252_g1_i2	Follistatin-related protein 5-like	LOC105454730	7.42	89	11
149	c72164_g1_i1	Aldehyde dehydrogenase, dimeric NADP-preferring	LOC105201109	7.19	63	8
150	c78495_g1_i1	Myosin-iiib-like	LOC105205193	7.16	55	7
151	c81945_g1_i1	Interleukin-1 receptor accessory protein-like 1	LOC105567535	7.08	62	8
152	c25801_g1_i1	POU domain, class 2, transcription factor 2-like	LOC105834934	7.01	1106	146
153	c84364_g1_i1	Leucine-rich repeat-containing protein 15-like	LOC105193718	6.94	1418	189
154	c87001_g6_i1	Endocuticle structural glycoprotein sgabd-8-like	LOC105193185	6.85	948	128

155	c78161_g1_i2	4-coumarate--coa ligase 1-like	LOC105196063	6.78	10063	1374
156	c79876_g1_i1	Proton-coupled amino acid transporter 2	LOC105208148	6.76	8259	1130
157	c86028_g1_i1	Solute carrier organic anion transporter family member 2A1	LOC105196351	6.73	168	23
158	c85681_g1_i1	Cytochrome P450 6a14	LOC105195645	6.71	24151	3329
159	c80124_g1_i1	Bipolar kinesin KRP-130-like	LOC105204079	6.69	102	14
160	c90337_g6_i3	Cationic amino acid transporter 2	LOC105833364	6.68	824	114
161	c84676_g1_i1	THAP domain-containing protein 4-like	LOC108749290	6.59	136	19
162	c68756_g1_i1	Facilitated trehalose transporter Tret1-like	LOC105827828	6.49	1011	144
163	c82982_g1_i2	Excitatory amino acid transporter 3	LOC105197539	6.44	864	124
164	c81272_g1_i2	Ras-related protein Rab-32	LOC105200376	6.41	1303	188
165	c83970_g1_i1	Serine/threonine-protein kinase SBK1	LOC105200885	6.38	497	72
166	c88370_g2_i1	Cytochrome P450 9e2-like	LOC105205219	6.34	658	96
167	c86095_g2_i2	Luciferin 4-monooxygenase-like	LOC105196065	6.33	240	35
168	c67432_g1_i1	Histone H2B	LOC105193510	6.3	130	19
169	c86028_g2_i1	Solute carrier organic anion transporter family member 2A1	LOC105196351	6.28	20939	3087
170	c63559_g1_i3	Synaptic vesicle glycoprotein 2C-like	LOC105202390	6.26	30583	4519
171	c83670_g2_i1	Lipase member H-B-like	LOC105207817	6.25	129	19
172	c80129_g1_i1	Zinc/iron permease	LOC105198488	6.18	648	97
173	c89754_g5_i1	Cytochrome P450 9e2-like	LOC105207597	6.12	199	30
174	c72067_g1_i1	Protein slit-like	LOC105193985	6.07	13223	2017
175	c84253_g1_i2	Nuclease HARBI1	LOC108766859	6.02	1230	189
176	c88518_g1_i4	Zinc transporter 1	LOC105828562	5.84	524	83
177	c74564_g1_i1	Protein N-terminal asparagine amidohydrolase	LOC105196325	5.8	239	38
178	c73090_g1_i1	TNF receptor-associated factor 5	LOC105193485	5.8	220	35
179	c88690_g6_i1	Maf-like protein CTC_02076	LOC106743984	5.8	314	50
180	c89324_g3_i3	Thiamine transporter 2-like	LOC105200122	5.72	953	154
181	c90227_g1_i3	Zinc finger protein	KIAA0543	5.72	291	47
182	c89082_g1_i1	Putative inorganic phosphate cotransporter	LOC105196548	5.71	334	54
183	c61818_g1_i1	Mucin-17-like	LOC105201236	5.71	161	26
184	c66989_g1_i4	Facilitated trehalose transporter Tret1	LOC105197057	5.69	2097	341
185	c87996_g2_i1	Argininosuccinate lyase	LOC105460741	5.62	626	103
186	c83659_g3_i1	Protein DDB_G0271670-like	LOC105196129	5.61	225	37

187	c89675_g1_i7	Phospholipid scramblase 1-like	LOC105202360	5.58	688	114
188	c88930_g1_i1	Limbic system-associated membrane protein	LOC105194273	5.5	280	47
189	c79982_g1_i2	Toll-like receptor 3	LOC105202230	5.49	297	50
190	c78499_g1_i2	Peptidoglycan-recognition protein 1-like	LOC105196158	5.39	635	109
191	c80006_g1_i4	Sodium-dependent multivitamin transporter	LOC105199060	5.38	640	110
192	c1274_g1_i1	Venom acid phosphatase Acph-1-like	LOC105197655	5.37	4584	790
193	c89329_g1_i2	Elongation of very long chain fatty acids protein AAEL008004-like	LOC105201316	5.34	6845	1185
194	c74492_g2_i1	Facilitated trehalose transporter Tret1-like	LOC105827828	5.33	1193	207
195	c3390_g1_i2	CD63 antigen-like	LOC105204097	5.32	874	152
196	c81964_g1_i1	Maf-like protein CTC_02076	LOC106743984	5.3	367	64
197	c89742_g1_i3	Cytosolic 10-formyltetrahydrofolate dehydrogenase	LOC105206141	5.3	4030	704
198	c63119_g2_i1	Flavin reductase (NADPH)	LOC105455278	5.19	253	45
199	c88979_g1_i1	Elongation of very long chain fatty acids protein	LOC108726870	5.17	604	108
200	c90592_g1_i4	Glutamate receptor ionotropic, delta-1-like	LOC105193770	5.12	410	74
201	c8852_g1_i1	Solute carrier family 52, riboflavin transporter, member 3-B-like	LOC105200141	5.09	605	110
202	c87231_g1_i2	Venom acid phosphatase Acph-1-like	LOC105205693	5.03	3950	727
203	c86249_g1_i3	Lipoprotein receptor adapter protein 1-like	LOC105195968	5	3144	582
204	c112580_g1_i1	Slit homolog 1 protein-like	LOC105833050	4.97	1199	223
205	c90821_g8_i3	Fatty acid synthase-like	LOC105208165	4.93	60617	11385
206	c30585_g1_i1	Solute carrier organic anion transporter family member 5A1	LOC105196352	4.92	10270	1931
207	c89832_g1_i1	RNA-directed DNA polymerase from mobile element jockey-like	LOC105205336	4.89	5484	1037
208	c39740_g1_i1	Elongation of very long chain fatty acids protein	LOC105207300	4.85	760	145
209	c68675_g1_i1	Transferrin-like	LOC105202693	4.79	17799	3439
210	c89805_g2_i2	Fatty acyl-coa reductase 1-like	LOC105201568	4.78	1225	237
211	c88936_g1_i1	Integral membrane protein GPR155	LOC105276468	4.75	1115	217
212	c83474_g1_i2	Peroxisomal hydratase-dehydrogenase-epimerase-like	LOC105197412	4.74	10103	1972
213	c84796_g1_i1	Vasorin-like	LOC105203944	4.73	3827	748
214	c79663_g1_i2	Lipopolysaccharide-induced tumor necrosis factor-alpha factor homolog	LOC105200998	4.67	3839	760
215	c89911_g3_i2	Cytochrome P450 9e2-like	LOC105207593	4.59	5843	1177

Table S8.

No	Contig	Gene	Accession number	Fold change	FPKM	
					T9	T25
1	c112558_g1_i1	actin-like protein	LOC105205816	-4.76	783	3447
2	c86185_g1_i1	ATP-binding cassette sub-family G member 8	LOC105197415	-4.9	776	3520
3	c88952_g2_i2	UNC93-like protein	LOC105208193	-4.96	75	345
4	c76554_g1_i2	calcium-independent phospholipase A2-gamma-like	LOC105203065	-4.99	8639	39909
5	c89852_g11_i1	blastopia polyprotein	LOC105194430	-5.06	49	230
6	c118149_g2_i1	ATP synthase 6	ADP01787.1	-5.15	2057	9806
7	c89016_g3_i1	capsid protein precursor	BAA32554.1	-5.22	95	459
8	c4730_g1_i2	pheromone-binding protein-related protein 6	ADX94407.1	-5.27	71	347
9	c89095_g2_i2	putative glycine-rich cell wall structural protein	LOC105199385	-5.36	35	174
10	c86197_g1_i2	anoctamin-4	LOC105199723	-5.39	262	1308
11	c90780_g1_i3	Peptidase_A17	LOC105457098	-5.5	51	260
12	c30447_g1_i1	putative mediator of RNA polymerase II transcription subunit 12	LOC105204368	-5.55	103	530
13	c74612_g1_i2	myosin heavy chain, muscle-like	PF01576.16	-5.73	54	287
14	c86618_g2_i1	forkhead box protein J1-A-like	LOC105196566	-5.79	18	97
15	c87519_g1_i2	sodium- and chloride-dependent GABA transporter 1-like	LOC105193795	-5.86	60	326
16	c74554_g2_i1	homeobox protein Nkx-2.4-like	LOC106740776	-5.89	52	284
17	c70844_g1_i1	general odorant-binding protein lush	LOC105199702	-5.91	63	345
18	c83852_g1_i2	elastin-like	LOC105197142	-6	39	217
19	c79559_g1_i1	BTB/POZ domain-containing protein KCTD16	LOC105839142	-6.01	29	162
20	c76451_g1_i1	vicilin-like seed storage protein	LOC105184175	-6.04	16	90
21	c102478_g1_i1	hormone-sensitive lipase	LOC105198580	-6.34	12	71
22	c84865_g1_i1	Phage_integrase	PF00589.19	-6.47	45	270
23	c65289_g1_i1	endocuticle structural glycoprotein SgAbd-8	LOC105192792	-6.47	46	276
24	c112589_g1_i1	general odorant-binding protein 72	LOC105193332	-6.52	572	3454
25	c87039_g1_i1	odorant receptor 43a-like	LOC105193356	-6.54	27	164
26	c89095_g1_i1	putative glycine-rich cell wall structural protein 1	LOC105199385	-6.6	50	306
27	c81505_g1_i1	ras-like protein 3	LOC105203745	-6.6	30	184

28	c77971_g1_i1	F-box only protein 32	LOC105835729	-6.68	34	211
29	c84617_g3_i3	probable H/ACA ribonucleoprotein complex subunit 1	LOC105196699	-7.04	18	118
30	c107361_g1_i1	elastin-like	LOC105197142	-7.31	41	278
31	c81007_g1_i1	leucine-rich repeat protein soc-2-like	LOC105831958	-7.46	8	56
32	c88118_g2_i1	peptidyl-prolyl cis-trans isomerase G-like	LOC105196526	-7.46	373	2576
33	c76977_g1_i5	odorant receptor 4-like	LOC105831255	-7.58	12	85
34	c49508_g5_i1	twitchin-like	LOC105195136	-7.65	29	206
35	c75673_g1_i1	ankyrin repeat domain-containing protein 63-like	LOC105199979	-8.33	10	78
36	c82673_g1_i1	cAMP-dependent protein kinase catalytic subunit beta-like	LOC105197741	-8.4	13	102
37	c85185_g1_i2	protein NPC2 homolog	LOC105197297	-8.76	56	455
38	c19617_g1_i1	odorant receptor 85f-like	LOC105834982	-8.96	7	59
39	c78787_g1_i1	hexamerin-like	LOC105204474	-8.96	24	200
40	c86715_g1_i2	odorant receptor 13a-like	LOC105830626	-9.05	8	68
41	c112576_g1_i1	general odorant-binding protein 69a-like	LOC105675856	-9.23	1751	14950
42	c63269_g1_i1	hypertrehalosaemic prohormone-like	LOC105203025	-9.54	6	54
43	c87416_g3_i1	cyclin-dependent kinase 5 activator 1	LOC105459892	-9.87	7	65
44	c73804_g1_i2	cytochrome P450 305a1	LOC105201474	-9.99	8	75
45	c10456_g1_i1	transmembrane protease serine 9-like	LOC105194707	-10.18	61	576
46	c107620_g1_i1	testis-specific serine/threonine-protein kinase 3	LOC105199628	-10.25	6	58
47	c82666_g1_i2	ryanodine receptor 44F	LOC105195231	-10.42	6	59
48	c81773_g1_i1	hexosaminidase D-like	LOC105202946	-10.48	7	69
49	c29304_g1_i1	electroneutral sodium bicarbonate exchanger 1	LOC105566242	-10.78	6	61
50	c86999_g1_i1	paired box protein Pax-6-like	LOC105200938	-10.94	23	234
51	c86029_g1_i1	RNA-binding protein 24-like	LOC105196353	-11.09	3	32
52	c90365_g3_i1	ATP-binding cassette sub-family G member 4	LOC105196997	-11.09	3	32
53	c102897_g1_i1	mutS protein homolog 4-like	LOC105205496	-11.09	3	32
54	c78848_g1_i1	Mariner Mos1 transposase	PNF25331.1	-11.18	8	84
55	c77575_g1_i1	odorant receptor 82a-like	LOC105195341	-11.44	3	33
56	c85725_g1_i2	claw keratin-like	LOC105197289	-11.45	8	86
57	c90623_g1_i1	aminopeptidase N	LOC105205293	-11.49	72	767
58	c88403_g1_i1	homeobox protein prospero	LOC105274940	-12.51	8	94
59	c96614_g1_i1	NADH dehydrogenase subunit 2	LOC9977801	-12.63	576	6731

60	c12936_g1_i1	synaptic vesicular amine transporter	LOC105200330	-12.66	5	60
61	c69128_g2_i2	UDP-glucuronosyltransferase 2B18-like	LOC105200783	-13.2	7	87
62	c86883_g1_i1	synaptic vesicular amine transporter	LOC105462413	-13.72	5	65
63	c47308_g1_i1	eye-specific diacylglycerol kinase	LOC105566924	-13.76	2	27
64	c79548_g1_i1	probable G-protein coupled receptor 158	LOC105153594	-13.85	3	40
65	c106955_g1_i1	ejaculatory bulb-specific protein 3	LOC105196182	-13.92	111	1431
66	c87936_g5_i1	chymotrypsin-1-like	LOC105199117	-14.07	19	249
67	c74191_g1_i1	transcriptional activator protein Pur-beta	LOC108726115	-14.27	2	28
68	c75659_g1_i1	octapeptide-repeat protein T2-like	LOC105194925	-14.27	2	28
69	c86657_g1_i1	putative golgin subfamily A member 6-like protein 6	LOC105667571	-14.68	4	56
70	c89345_g8_i1	odorant receptor coreceptor	LOC105831208	-15.31	20	285
71	c90619_g1_i1	sensory neuron membrane protein 1-like	LOC105195544	-15.47	111	1591
72	c115467_g1_i1	odorant receptor 85c-like	LOC105618159	-16.8	2	33
73	c114061_g1_i1	talin-1	LOC105450889	-17.47	6	99
74	c79106_g1_i2	phospholipase A1-like	LOC105205771	-17.56	16	262
75	c77963_g2_i1	laccase-3-like	LOC105557022	17.82	2	35
76	c83165_g1_i1	glucose dehydrogenase [FAD, quinone]-like	LOC105459617	-19.85	2	39
77	c84617_g2_i1	H/ACA ribonucleoprotein complex subunit 1	LOC105196699	-19.92	4	76
78	c90059_g3_i1	putative golgin subfamily A member 6-like protein 6	LOC105667571	-21.16	1	22
79	c64736_g1_i1	odorant receptor 43a-like	LOC105150515	-21.16	1	22
80	c13543_g2_i1	NADH dehydrogenase subunit 6 (mitochondrion)	CEF49550.1	-21.41	35	696
81	c104660_g1_i1	peripheral plasma membrane protein CASK	LOC105833113	-22.39	2	44
82	c72825_g1_i1	arylphorin subunit alpha-like	LOC105192898	-23.02	18	386
83	c94540_g1_i1	heat shock 70kDa protein 8 isoform 2	PF00012	-23.07	1	24
84	c116148_g1_i1	neutral ceramidase-like	LOC105197211	-24.03	1	25
85	c93265_g1_i1	serpin B3-like	LOC105193420	-24.03	1	25
86	c79569_g1_i1	talin-1, transcript variant X4	LOC105450889	-24.87	9	210
87	c70254_g1_i1	spidroin-1-like	LOC105202339	-25.18	56	1308
88	c90934_g1_i1	NADH dehydrogenase subunit 4L	ADP01792.1	-25.37	17	402
89	c81593_g1_i2	odorant receptor 43a-like isoform X1	PF02949.17	-25.94	1	27
90	c91662_g1_i1	neurotrimin-like	LOC105836374	-25.94	1	27
91	c113272_g1_i1	homeobox protein prophet of Pit-1	LOC105834870	-26.9	1	28

92	c73776_g1_i3	odorant receptor 13a-like	LOC105193517	-26.9	1	28
93	c75712_g2_i1	zinc finger protein 236-like	LOC108721033	-27.85	1	29
94	c90181_g1_i1	sensory neuron membrane protein 1-like	LOC105195545	-31.24	7	206
95	c48237_g1_i1	protein NYNRIN-like	LOC105199732	-31.68	1	33
96	c26648_g1_i1	keratinocyte proline-rich protein-like	LOC105196700	-33.51	8	252
97	c74923_g1_i1	peptidylprolyl isomerase A	LOC268373	-33.59	1	35
98	c85316_g3_i2	peritrophin-48-like	LOC105203677	-34.78	5	165
99	c72862_g1_i1	tetra-peptide repeat homeobox protein 1-like	LOC105202337	-35.26	3	102
100	c57908_g1_i1	talin-2-like isoform X1	LOC107359463	-35.5	1	37
101	c78623_g1_i1	homeobox protein prophet of Pit-1	LOC105834870	-38.37	1	40
102	c90572_g1_i6	odorant receptor 43a-like	LOC105198227	-44.11	1	46
103	c82822_g1_i1	NADH dehydrogenase subunit 5	LOC17721	-54.35	2	107
104	c71522_g1_i1	NADH dehydrogenase subunit 2	LOC17717	-55.59	1	58
105	c40490_g1_i1	protein G12-like	LOC105201881	-61.67	27	1548
106	c82068_g1_i1	chitinase-3-like protein 1	LOC105204966	-66.23	4	253
107	c89345_g3_i1	odorant receptor coreceptor	LOC105424270	-93.04	10	872
108	c82058_g1_i1	chymotrypsin-1-like	LOC105199115	-101.54	2	200
109	c80587_g1_i1	NADH-quinone oxidoreductase chain 13	AFX73489.1	-103.41	1	108
110	c84900_g2_i1	elongation factor 1-alpha 1-like isoform X1	LOC105613550	-106.1	2	209
111	c90598_g2_i3	heat shock protein	PF00012	-115.85	1	121
112	c61759_g1_i1	NADH dehydrogenase subunit 1	AFX73545.1	-128.28	1	134
113	c90390_g2_i2	YEATS domain-containing protein 2-like	LOC105677732	-203.84	1	213
114	c34823_g1_i1	ATP synthase subunit A	AFX73615.1	-357.83	1	374
115	c13550_g1_i1	polyprotein	AQX17788.1	-1024.48	1	1071

Table S9.

Contig	Unigene	KEGG number	Enriched KEGG pathways
c17281_g1_i1	Chymotrypsin-2-like	105200925	Neuroactive ligand-receptor interaction
c102119_g1_i1	Chymotrypsin-1-like	105193305	Neuroactive ligand-receptor interaction
c75529_g1_i1	Alpha-glucosidase-like	105193841	Galactase metabolism
			Metabolic Pathways
			Starch and sucrose metabolism
c73740_g2_i1	Alpha-glucosidase-like	105672537	Galactase metabolism
			Metabolic Pathways
			Starch and sucrose metabolism
c89980_g2_i1	Fatty acyl-coa reductase 1	105200177	Peroxisome
c88786_g3_i1	Transmembrane protease serine 9-like	105200924	Neuroactive ligand-receptor interaction
c90634_g3_i2	Fatty acyl-coa reductase 1-like	105147980	Peroxisome
c80199_g1_i1	Alpha-glucosidase-like	105193841	Galactase metabolism
			Metabolic Pathways
			Starch and sucrose metabolism
c80341_g1_i1	Gamma-aminobutyric acid receptor alpha-like	105433700	Neuroactive ligand-receptor interaction
c400319_g1_i1	Alpha-glucosidase-like	105193841	Ubiquinone and other terpenoid-quinone biosynthesis
			Metabolic pathway
c90634_g5_i1	Fatty acyl-coa reductase 1-like	105147980	Peroxisome
c88786_g2_i2	Chymotrypsin-2-like	105200925	Neuroactive ligand-receptor interaction
c80784_g2_i1	Serine protease 53-like	105200928	Neuroactive ligand-receptor interaction
c84339_g1_i1	Chymotrypsin-2-like	105193273	Neuroactive ligand-receptor interaction
c88504_g1_i1	Uridine 5'-monophosphate synthase-like	105201038	Drug metabolism - other enzymes
			Metabolic Pathways
			Pyrimidine metabolism
c114158_g1_i1	Cysteine sulfinic acid decarboxylase	105202384	Metabolic Pathways
			Taurine and hypotaurine metabolism
c89980_g1_i1	Fatty acyl-coa reductase 1	105200177	Peroxisome
c90297_g13_i1	Inositol-trisphosphate 3-kinase A	105199246	Inositol phosphate metabolism
			Metabolic Pathways
			Phosphatidylinositol signaling system
c119320_g1_i1	4-coumarate--coa ligase 1-like	105196063	Metabolic Pathways
			Ubiquinone and other terpenoid-quinone biosynthesis
c66823_g1_i1	Chymotrypsin-like protease CTRL-1	105206213	Neuroactive ligand-receptor interaction
c78680_g1_i1	Pancreatic triacylglycerol lipase-like	105199576	Glycerolipid metabolism
			Metabolic Pathways
c113360_g1_i1	Elongation of very long chain fatty acids protein 7-like	105207261	Biosynthesis of unsaturated fatty acids
			Fatty acid elongation
			Fatty acid metabolism
			Metabolic Pathways
c77768_g1_i1	Ornithine decarboxylase 2-like	105200774	Arginine and proline metabolism
			Glutathione metabolism
			Metabolic Pathways
c77048_g1_i1	Peptidoglycan-recognition protein 1-like	105196158	Toll and Imd signaling pathway
c87774_g2_i1	Fatty acid synthase-like	108748804	Fatty acid biosynthesis
			Fatty acid metabolism
			Metabolic Pathways
c89947_g1_i2	Lipase 3-like	105208099	Lysosome
			Steroid biosynthesis
c83670_g1_i1	Lipase member H-B-like	105207817	Glycerolipid metabolism
			Metabolic Pathways
c75351_g1_i2	Trypsin-1-like	105200778	Neuroactive ligand-receptor interaction
c88751_g1_i2	Acyl-coa Delta(11) desaturase	105200008	Fatty acid metabolism
			Glycerolipid metabolism
			Metabolic Pathways
c90816_g2_i2	Caspase-1-like	105198123	Apoptosis - fly
			Apoptosis - multiple species
c21111_g1_i1	Fatty acid synthase	105145528	Fatty acid biosynthesis
			Fatty acid metabolism
			Metabolic Pathways
c76996_g1_i2	Acyl-coa Delta(11) desaturase-like	105194093	Biosynthesis of unsaturated fatty acids

			Fatty acid metabolism<br>Metabolic Pathways
c88025_g3_i1	Putative fatty acyl-coa reductase CG5065	105203556	Peroxisome
c83419_g3_i2	Fatty acid synthase-like	105196297	Fatty acid biosynthesis<br>Fatty acid metabolism<br>Metabolic Pathways
c88979_g2_i4	Elongation of very long chain fatty acids protein AAEL008004-like	105201316	Biosynthesis of unsaturated fatty acids<br>Fatty acid elongation<br>Fatty acid metabolism<br>Metabolic Pathways
c11645_g1_i1	Anionic trypsin-2-like	105207761	Neuroactive ligand-receptor interaction
c88979_g4_i2	Elongation of very long chain fatty acids protein 7-like	105201228	Biosynthesis of unsaturated fatty acids<br>Fatty acid elongation<br>Fatty acid metabolism<br>Metabolic Pathways
c54236_g1_i1	Caspase-1-like	105193867	Apoptosis - fly<br>Apoptosis - multiple species
c108287_g1_i1	Peptidoglycan-recognition protein SC2-like	105564513	Toll and Imd signaling pathway
c80784_g1_i1	Trypsin-1-like	105198898	Neuroactive ligand-receptor interaction
c71252_g1_i2	Follistatin-related protein 5-like	105454730	Biosynthesis of unsaturated fatty acids<br>Fatty acid metabolism<br>Metabolic Pathways
c78495_g1_i1	Myosin-iiib-like	105205193	Phtotransduction-fly
c86095_g2_i2	Luciferin 4-monooxygenase-like	105196065	Metabolic Pathways<br>Ubiquinone and other terpenoid-quinone biosynthesis
c63559_g1_i3	Synaptic vesicle glycoprotein 2C-like	105202390	ECM-receptor interaction
c61818_g1_i1	Mucin-17-like	105201236	Metabolic Pathways<br>Amino sugar and nucleotide sugar metabolism
c87996_g2_i1	Argininosuccinate lyase	105201228	Alanine, aspartate and glutamate metabolism<br>Arginine biosynthesis<br>Biosynthesis of amino acids<br>Metabolic Pathways
c78499_g1_i2	Peptidoglycan-recognition protein 1-like	105196158	Toll and Imd signaling pathway
c89742_g1_i3	Cytosolic 10-formyltetrahydrofolate dehydrogenase	105206141	One carbon pool by folate
c63119_g2_i1	Flavin reductase (NADPH)-like	105203453	Porphyrin and chlorophyll metabolism<br>Riboflavin metabolism<br>Metabolic Pathways
c86249_g1_i3	Low density lipoprotein receptor adapter protein 1-like	105253065	Endocytosis
c89805_g2_i2	Fatty acyl-coa reductase 1-like	105201568	Peroxisome
c79663_g1_i2	Lipopolysaccharide-induced tumor necrosis factor-alpha factor homolog	105426094	Lysosome
c77971_g1_i1	F-box only protein 32	105835729	FoxO signaling pathway
c82673_g1_i1	Camp-dependent protein kinase catalytic subunit beta-like	105197741	Autophagy - animal<br>Hedgehog signaling pathway - fly<br>Longevity regulating pathway - multiple species<br>Wnt signaling pathway
c85185_g1_i2	Protein NPC2 homolog	105197297	Lysosome
c73804_g1_i2	Probable cytochrome P450 305a1	105201474	Insect hormone biosynthesis
c81773_g1_i1	Hexosaminidase D-like	105202946	Other glycan degradation<br>Various typees of N-glycan biosynthesis<br>Metabolic Pathways
c90365_g3_i1	ATP-binding cassette sub-family G member 4	105196997	ABC transporters
c88403_g1_i1	Homeobox protein prospero	105274940	MAPK signaling pathway - fly
c96614_g1_i1	NADH dehydrogenase subunit 2	9977801	Oxidative phosphorylation<br>Metabolic Pathways
c69128_g2_i2	UDP-glucuronosyltransferase 2B18-like	105200783	Ascorbate and aldarate metabolism<br>Drug metabolism - cytochrome P450<br>Drug metabolism - other enzymes<br>Metabolic Pathways<br>Metabolism of xenobiotics by cytochrome P450<br>Pentose and glucuronate interconversions<br>Porphyrin and chlorophyll metabolism<br>Retinol metabolism
c47308_g1_i1	Eye-specific diacylglycerol kinase	105566924	Glycerolipid metabolism<br>Glycerophospholipid metabolism<br>Metabolic Pathways

			Phosphatidylinositol signaling system
c87936_g5_i1	Chymotrypsin-1-like	105199117	Neuroactive ligand-receptor interaction
c88935_g4_i1	Histone-lysine N-methyltransferase SETMAR-like	105247966	Lysine degradation
			Metabolism of xenobiotics by cytochrome P450
c116148_g1_i1	Neutral ceramidase-like	105197211	Sphingolipid metabolism
			Metabolic Pathways
c90934_g1_i1	NADH dehydrogenase subunit 4L	9977797	Oxidative phosphorylation
			Metabolic Pathways
c90181_g1_i1	Sensory neuron membrane protein 1-like	105195545	Phagosome
c85316_g3_i2	Peritrophin-48-like	105203677	DNA replication
c90934_g1_i1	Chitinase-3-like protein 1	9977797	Amino sugar and nucleotide sugar metabolism
			Metabolic Pathways
c82058_g1_i1	Chymotrypsin-1-like	105199115	Neuroactive ligand-receptor interaction
c87346_g2_i1	Phospholip_A2	105203257	alpha-Linoleic acid metabolism
			Arachidonic acid metabolism
			Glycerophospholipid metabolism
			Metabolic Pathways
			Ether lipid metabolism
			Linoleic acid metabolism
c90623_g1_i1	Aminopeptidase N	105205293	Glutathione metabolism
			Metabolic Pathways
c82068_g1_i1	Chitinase-3-like protein 1	105204966	Amino sugar and nucleotide sugar metabolism
			Metabolic Pathways
c90821_g8_i3	Fatty acid synthase-like	105208165	Fatty acid biosynthesis
			Fatty acid metabolism
			Metabolic Pathways
c47308_g1_i1	elongation of very long chain fatty acids protein 7-like	105201228	Biosynthesis of unsaturated fatty acids
			Fatty acid elongation
			Metabolic Pathways
			Fatty acid metabolism
c78161_g1_i2	4-coumarate--CoA ligase 1-like	105196063	Ubiquinone and other terpenoid-quinone biosynthesis
			Metabolic Pathways
c76863_g1_i2	4-coumarate--CoA ligase 1-like	105196063	Ubiquinone and other terpenoid-quinone biosynthesis
			Metabolic Pathways
c32213_g1_i1	elongation of very long chain fatty acids protein 7-like	LOC105829 987	Biosynthesis of unsaturated fatty acids
			Fatty acid elongation
			Metabolic Pathways
			Fatty acid metabolism
c88709_g1_i1	Uncharacterized	105205293	Glutathione metabolism
			Metabolic Pathways

Table S10.

GO term	Unigenes	DEGs (FC≥2, FC≤-2)
**Biological process**		
Reproduction	90 (0.17%*)	6 (1.24%**)
Obsolete protein import into nucleus, docking	18 (0.03%)	0 (0%)
Obsolete MAPK import into nucleus	1 (0%)	0 (0%)
Cell killing	10 (0.02%)	0 (0%)
Immune system process	506 (0.94%)	2 (0.41%)
Obsolete snRNP protein import into nucleus	1 (0%)	0 (0%)
Obsolete negative regulation of transcription from RNA polymerase II	5 (0.01%)	0 (0%)
Behavior	784 (1.45%)	12 (2.48%)
Metabolic process	9,319 (17.22%)	110 (22.73%)
Cell proliferation	351 (0.65%)	3 (0.62%)
Cellular process	13,159 (24.32%)	109 (22.52%)
Obsolete storage protein import into fat body	2 (0%)	0 (0%)
Carbon utilization	1 (0%)	0 (0%)
Obsolete molecular hydrogen transport	1 (0%)	0 (0%)

Nitrogen utilization	11 (0.02)	0 (0%)
Reproductive process	1,414 (2.61%)	10 (2.07%)
Biological adhesion	476 (0.88%)	2 (0.41%)
Signaling	585 (1.08%)	3 (0.62%)
Multicellular organismal process	2,561 (4.73%)	30 (6.2%)
Developmental process	3,838 (7.09%)	23 (4.75%)
Obsolete suppression by virus of host RIG-I activity by RIG-I proteolysis	18 (0.03%)	5 (1.03%)
Obsolete suppression by virus of host MAVS activity by MAVS proteolysis	11 (0.02%)	3 (0.62%)
Growth	387 (0.72%)	1 (0.21%)
Locomotion	732 (1.35%)	3 (0.62%)
Obsolete transcription factor import into nucleus	2 (0%)	0 (0%)
Obsolete negative regulation of transcription factor import into nucleus	1 (0%)	0 (0%)
Obsolete positive regulation of transcription factor import into nucleus	16 (0.03%)	0 (0%)
Pigmentation	70 (0.13%)	0 (0%)
Obsolete mycelium development	5 (0.01%)	0 (0%)
Obsolete RNA polymerase II complex import to nucleus	1 (0%)	0 (0%)
Biological phase	14 (0.03%)	0 (0%)
Obsolete intermembrane transport	1 (0%)	0 (0%)
Rhythmic process	172 (0.32%)	4 (0.83%)
Response to stimulus	2,975 (5.5%)	33 (6.82%)
Localization	3,515 (6.5%)	36 (7.44%)
Obsolete negative regulation of ubiquitin-protein ligase activity	2 (0%)	0 (0%)
Multi-organism process	566 (1.05%)	11 (2.27%)
Biological regulation	8,026 (14.83%)	52 (10.74%)
Cellular component organization or biogenesis	4,240 (7.84%)	23 (4.75%)
Obsolete mitochondrial respiratory chain complex III biogenesis	1 (0%)	0 (0%)
Cell aggregation	5 (0.01%)	0 (0%)
Detoxification	85 (0.16%)	3 (0.62%)
Presynaptic process involved in chemical synaptic transmission	127 (0.23%)	0 (0%)
Obsolete RNA polymerase III complex import into nucleus	1 (0%)	0 (0%)
**Cellular component**		
Extracellular region	530 (1.15%)	15 (3.51%)
Cell	42 (0.09%)	1 (0.23%)
Nucleoid	27 (0.06%)	0 (0%)
Membrane	3,978 (8.6%)	55 (12.88%)
Virion	3 (0.01%)	0 (0%)
Cell junction	663 (1.43%)	5 (1.17%)
Membrane-enclosed lumen	270 (0.58%)	0 (0%)
Protein-containing complex	5,210 (11.26%)	26 (6.09%)
Organelle	7,513 (16.24%)	45 (10.54%)
Other organism part	5 (0.01%)	0 (0%)
Extracellular region part	1,568 (3.39%)	30 (7.03%)
Organelle part	6,190 (13.38%)	45 (10.54%)
Virion part	4 (0.01%)	0 (0%)
Membrane part	4,536 (9.8%)	65 (15.22%)
Synapse part	387 (0.84%)	(0.94%)
Cell part	14,694 (31.76%)	132 (30.91%)
Synapse	290 (0.63%)	2 (0.47%)
Obsolete MLL5-L complex	3 (0.01%)	0 (0%)
Supramolecular complex	350 (0.76%)	2 (0.47%)
**Molecular function**		
Obsolete RNA polymerase III type 3 promoter TFIIIB-type transcription factor	3 (0.01%)	0 (0%)

Catalytic activity	8,531 (34.38%)	124 (42.47%)
Obsolete gamma-glutamyltransferase activity	3 (0.01%)	0 (0%)
Signal transducer activity	1,063 (4.28%)	14 (4.79%)
Structural molecule activity	1,023 (4.12%)	8 (2.74%)
Transporter activity	1,565 (6.31%)	36 (12.33%)
Binding	10,355 (41.73%)	97 (33.22%)
Antioxidant activity	92 (0.37%)	0 (0%)
Protein tag	27 (0.11%)	0 (0%)
Cargo receptor activity	41 (0.17%)	1 (0.34%)
Translation regulator activity	31 (0.12%)	0 (0%)
Obsolete cyclic pyranopterin monophosphate synthase activity	3 (0.01%)	0 (0%)
Molecular function regulator	937 (3.78%)	5 (1.71%)
Hijacked molecular function	8 (0.03%)	0 (0%)
Molecular carrier activity	31 (0.12%)	0 (0%)
Transcription regulator activity	1,100 (4.43%)	7 (2.4%)
